# Exposure of preterm neonates receiving total parenteral nutrition to phthalates and its impact on neurodevelopment at the age of 2 months

**DOI:** 10.1038/s41598-023-33715-w

**Published:** 2023-04-28

**Authors:** Iman Al-Saleh, Rola Elkhatib, Hissah Alnuwaysir, Hesham Aldhalaan, Eiman Alismail, Abdulaziz Binmanee, Amal Hawari, Fahad Alhazzani, Mohammad Bin Jabr, Gamal Mohamed

**Affiliations:** 1 King Faisal Specialist Hospital and Research Centre, Environmental Health Program (MBC#03), P.O. Box: 3354, Riyadh, 11211 Saudi Arabia; 2 Center for Autism Research, King Faisal Specialist Hospital and Research Centre, P.O. Box: 3354, Riyadh, 11211 Saudi Arabia; 3 Neonatal Critical Care Section, Department of Pediatrics, King Faisal Specialist Hospital and Research Centre, P.O. Box: 3354, Riyadh, 11211 Saudi Arabia; 4 Biostatistics, Epidemiology and Scientific Computing Department, King Faisal Specialist Hospital and Research Centre, P.O. Box: 3354, Riyadh, 11211 Saudi Arabia

**Keywords:** Risk factors, Environmental impact, Medical research, Paediatric research

## Abstract

This prospective study assessed the exposure to phthalates of preterm neonates who received total parenteral nutrition (TPN) during their stay in the neonatal intensive care unit (NICU) and the risk of neurodevelopment delays at the age of 2 months. Our study recruited 33 preterm neonates who required TPN upon NICU admission. Urine samples for analyzing phthalate metabolites were obtained at admission and then daily until the last day of receiving TPN. Phthalates in the daily TPN received by the preterm neonates were analyzed. The neurodevelopment of the neonates was assessed using the Ages and Stages Questionnaire Edition 3 (ASQ-3). Diethyl phthalate and butyl benzyl phthalate were found in all TPN samples, while 27% and 83% contained dibutyl phthalate and di-(2-ethylhexyl) phthalate (DEHP), respectively. Yet, the daily dose of each phthalate that our preterm neonates received from TPN was much lower than the recommended tolerable limit. Urinary levels of monobenzyl phthalate and four metabolites of DEHP [i.e., mono-(2-ethylhexyl) phthalate (MEHP), mono-(2-ethyl-5-hydroxyhexyl) phthalate, mono-(2-ethyl-5-oxohexyl) phthalate (MEOHP), and mono-(2-ethyl-5-carboxypentyl) phthalate (MECPP)] and the sum of four DEHP metabolites (∑_4_DEHP) increased significantly in preterm neonates before discharge. However, these levels were not correlated with their phthalate parent compounds in TPN, suggesting other sources of exposure in the NICU. At 2 months, we found that urinary levels of mono-iso-butyl phthalate (M*i*BP), MECPP, MEHP, and ∑_4_DEHP were inversely related to fine motor skills. After adjusting for head circumference, the inverse relationships remained significant, suggesting direct effects from phthalates. Given the extreme vulnerability of our population, it is critical to minimize exposure to phthalates during their NICU stay.

## Introduction

Premature neonates are those born before 37 weeks of pregnancy due to low birth weight < 5.5 pounds (2500 g) or a medical condition requiring specialized medical care. According to the World Health Organization, preterm birth complications are the leading cause of death among children under 5 years and were responsible for approximately 1 million deaths in 2015^1^. Additionally, premature neonates are prone to health complications later in life, such as respiratory diseases^2^, cardiovascular problems^3^, renal dysfunction^4^, and neurodevelopment delays^5^, and have a potentially higher risk of specific cancers^6^.

Total parenteral nutrition (TPN) has been an integral part of the clinical management of low birth weight premature neonates, as feeding via the enteral route is not attainable^7,8^. Studies have reported the risks to premature neonates from TPN solutions contaminated with chemicals and/or microbes^9,10^. Preterm neonates in a neonatal intensive care unit (NICU) setting are highly vulnerable to harmful chemicals, which can impact their growth and neurodevelopment in the long term^11^, and the authors suggest improving the NICU environment.

Phthalates can be another significant source of contamination of TPN solutions because they are used to soften plastics and thus are widely utilized in medical devices, primarily in bags and infusion tubing for intravenous solutions and feeding^12–15^. As phthalates are not chemically bound to the plastic matrix, they leach out and migrate into the air, food, or other materials^13^. Among phthalates, di (2-ehylhexyl) phthalate (DEHP) is the most widely used plasticizer in medical devices^16^, and may contribute to chronic complications, particularly in preterm infants^17^. Although alternative DEHP-free devices have been introduced, studies have found that a wide range of plasticizers, in addition to DEHP, are still detected in medical devices used in pediatric units^18^, including the NICU^19^. Following exposure, phthalates are rapidly metabolized to their corresponding hydrolytic monesters, which can be further transformed to more hydrophobic oxidative products, conjugated to glucuronic acid via enzymatic hydrolysis, and eliminated in the urine^20,21^. The glucuronidation process facilitates the urinary excretion of phthalates, reducing their potential toxicity^21^. Newborns and preterm infants have immature glucuronidation pathways, leading to slower urinary excretion of phthalates compared with older children and adults^22^. High phthalate levels in urine were reported in the first weeks of intensive care, following invasive procedures, and in preterm infants with a birth weight < 1000 g^23^. Infants with low birth weight and those diagnosed with septicemia or bronchopulmonary dysplasia had higher urinary phthalate levels because of prolonged exposure to medical equipment containing phthalates^24^. A limited number of studies have explored the relationship between exposure to some phthalates and neurobehavioral outcomes^25–28^. However, the results are still controversial. The potential toxicity of phthalates to infants in the NICU is a concern because of their small body size and health status^29^.

There is limited evidence on the exposure of preterm neonates in the NICU to contaminants through TPN solutions and their potential impact on neurologic development. The present study aimed to examine: (a) the contribution of TPN solutions contaminated with phthalate to the overall phthalate exposure of preterm neonates during their NICU stay and (b) the risk of neurodevelopmental delays at 2 months.

## Patients and methods

### Study design

**Figure 1:**
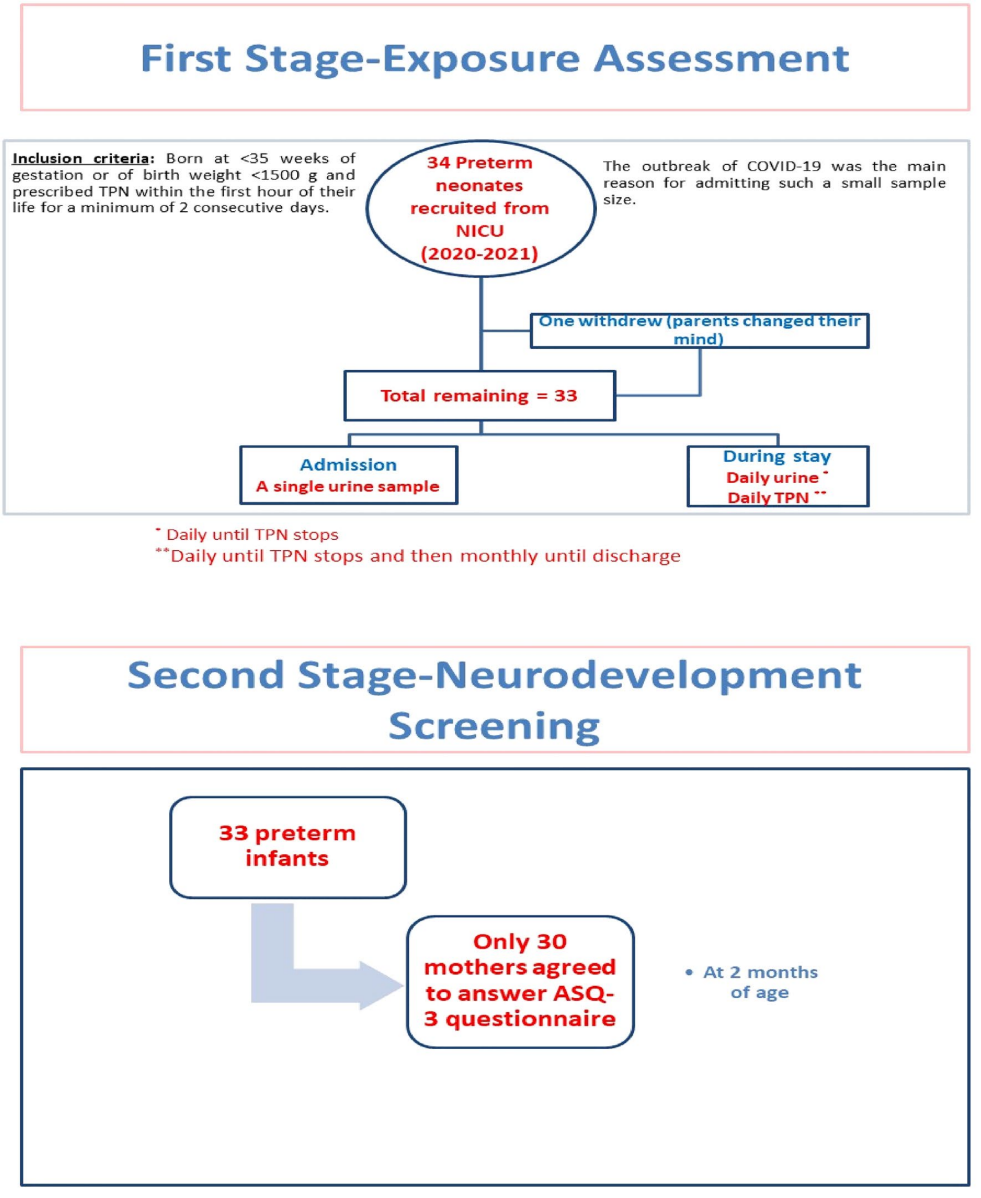
Flow chart of the study population.

A prospective cohort study that included 33 premature neonates admitted to the NICU at King Faisal Specialist Hospital & Research Centre (KFSH&RC) was conducted between January 2020 and March 2021. The inclusion criteria were preterm neonates born at < 35 weeks of gestation or of birth weight < 1500 g and prescribed TPN within the first hour of their life for a minimum of 2 consecutive days. Preterm neonates who received a vaccination or were prenatally diagnosed with genetic or metabolic disorders or gastrointestinal genetic anomalies were excluded. This study was conducted after obtaining the approval of the Research Ethics Committee of the KFSH&RC (RAC#2191092). The study details were explained to the mothers, and their written consent was obtained on behalf of the children. Participants were encouraged to ask questions about the study to ensure their understanding. The demographics (gender, gestational age), baseline measurements (body length and weight), and general health information of the studied infants at delivery and during their stay at NICU were gathered from the patient chart. The TPN solution administered during the study period was Olimel N9E^®^; SmofKabiven^®^ (Olimel; Baxter, Lessines, Belgium), the components of which were adjusted and individualized according to the patient's clinical condition. It contains fixed concentrations of trace elements (copper, zinc, manganese, selenium, sodium fluoride, and potassium chloride) at 0.1 mL/kg body weight. Figure 1 shows the data for the study population. Study quality was assessed using the STROBE template for observational studies^30^ (Table S1).

All experiments and methods were performed in accordance with relevant guidelines and regulations.

Throughout the manuscript, "admission" refers to samples collected from preterm neonates at admission before receiving TPN solutions. "Before discharge" refers to samples (daily for urine) collected from preterm neonates after receiving TPN solutions.

### Biological materials

Urine samples (1–2 mL) were collected at admission and then daily until TPN was discontinued, by applying a sterile adhesive plastic bag to their perineum. Urine was transferred using 10 mL plastic syringes into 10 mL glass screw-capped tubes and stored at − 30 °C until the analysis of phthalates. Aliquots of 100 µL were collected into 0.5 mL microcentrifuge Eppendorf tubes and stored at − 30 °C until the analysis of creatinine.

### TPN solutions

Leftover TPN solution was collected daily from each discarded bag received by a preterm neonate and aliquoted immediately into glass tubes. Both were stored at − 20 °C until analysis. For logistic reasons, phthalate compounds were measured in 195 samples instead of 230.

### Analytical techniques

#### Phthalate assessment of urine (metabolites) and TPN (parent compounds)

The levels of eight phthalate metabolites (µg/L), monoethyl phthalate (MEP), mono-*n*-butyl phthalate (M*n*BP), mono-iso-butyl phthalate (M*i*BP), mono-benzyl phthalate (MB*z*P), mono-(2-ethylhexyl) phthalate (MEHP), mono-(2-ethyl-5-hydroxyhexyl) phthalate (MEHHP), mono-(2-ethyl-5-oxohexyl) phthalate (MEOHP), and mono-(2-ethyl-5-carboxypentyl) phthalate (MECPP) were measured in urine samples using ultra-performance liquid chromatography and tandem mass spectrometry (Waters, Milford, USA), based on a modified method from previously published studies^31–33^. These metabolites were associated with five parental phthalates. MEP is a metabolite of diethyl phthalate (DEP), M*n*BP and M*i*BP are metabolites of dibutyl phthalate (DBP), MB*z*P is a metabolite of butyl benzyl phthalate (BB*z*P), and MECPP, MEHP, MEOHP, and MEHHP are metabolites of DEHP. The method detection limits (MDLs) for MEP, M*i*BP, M*n*BP, MB*z*P, MECPP, MEHHP, MEOHP, and MEHP were 1.7, 1.51, 1.76, 0.99, 1.57, 1.62, 0.61, and 2.07 µg/L, respectively. The accuracy of the metabolite measurements (except MEP) was assessed using the German External Quality Assessment Scheme. We also used internal quality control by spiking urine with known concentrations of the metabolites. The recovery of each metabolite in urine spiked with three concentrations (15, 80, and 150 µg/L) was 94–103%. Each run included blanks, quality controls, matrix-spiked samples, and unknown samples. The analytical procedures and method validation are presented in the Supplemental Material (Tables S2–S5).

DEP, DBP, BB*z*P, and DEHP in TPN solutions were analyzed using solid-phase microextraction (SPME) followed by gas chromatography/mass spectrometry (GC–MS). The calibration curves showed good linearity within the given concentration ranges of 0.5–16 µg/L for DEP, DBP, BB*z*P, and DEHP, with *r*^2^ > 0. 997. The MDLs for DEP, DBP, BB*z*P, and DEHP in µg/L were 0.05, 0.047, 0.169, and 0.05, respectively. The recoveries for both ranges were good. The relative standard deviations (RSDs) for within- and between-run precision were < 10%. Therefore, the SPME–GC–MS method exhibited good precision and accuracy for quantifying the six selected phthalate compounds in the TPN samples. The validation parameters for the analytical methods are presented in Tables S6 and S7 (Supplemental Material).

Molar sum of DEHP metabolites (∑_4_DEHP) was calculated by summing the ratio of each metabolite to its molecular weight: [MEHP/278.34] + [MEHHP/294.34] + [MECPP/308.33] + [MEOHP/292.33]. ∑_3_DEHP was calculated similarly but without MEHP. The molar concentrations of two DBP metabolites was calculated as [M*i*BP/222.24 + M*n*BP/222.24] and expressed as ∑DBP.

#### Other measurements

The urine samples for neonates were analyzed for creatinine (creat) to adjust for the effect of urinary flow rate on the urinary excretion of tested analytes^34^, presenting the results as micrograms of phthalate monoester per gram of urinary creatinine (µg/g creat).

### Neurodevelopment screening tools

After NICU discharge, 33 preterm neonates were scheduled for a 2-month follow-up visit at the Center for Autism Research at KFSH&RC during which the Ages and Stages Questionnaire 3 (ASQ-3) was conducted to identify whether the infant was at risk of delays in neurological development. Age was corrected for prematurity using the following link: Calculator: Add to or subtract from a date (timeanddate.com). The median corrected age at assessment was 4.2 months. The test is a comprehensive parent checklist, standardized for children between the ages of 1 and 66 months, that consists of 30 questions covering five domains of development: communication, gross motor, fine motor, problem-solving, and personal-social^35^. Each domain has a set of six items. The response of the parent to each item is yes (score = 10), sometimes (score = 5), or not yet (score = 0). The questionnaire takes 10–15 min for parents to complete. A total of 30 mothers agreed to answer the AQS-3 questionnaire.

### Statistical analysis

The characteristics of the preterm neonates were described using means with standard deviations (SDs) or medians (minimum–maximum) for continuous variables, or numbers (%) for categorical variables. Geometric means were calculated to describe the distributions of tested analytes that were logarithmically transformed to approximate a normal distribution. Values below the MDL were replaced with ½ MDL to provide unbiased estimates for the mean and SD, particularly with a small proportion of readings below MDL^36^. However, this approach might be of concern when more than 50% of the data are below MDL^37^. This method has been adopted by many researchers^38,39^. As shown in the Results section, a small proportion of phthalate metabolites measurements in the current study were < MDL, suggesting that an unbiased estimate of the relationship between exposure and the neurodevelopment outcome was provided^40^. Spearman rank correlation analysis (rs) was performed to test for associations between pairs of continuous variables. Mann–Whitney test was used for categorical variables. Using mixed-effects models, we calculated the variability in the levels of the phthalate metabolites measured in 13 urine samples of preterm neonates by estimating interclass correlation coefficients (ICCs) and their 95% confidence intervals (95%CIs). Reproducibility based on the ICCs was categorized as excellent (> 0.75), moderate (0.4 to 0.75), or weak (< 0.4)^41^. Generalized estimating equations (GEE) with Bonferroni adjustments were conducted to account for the correlation among repeated measuresSeparate multiple linear regression models were built for each neurodevelopmental score as a continuous dependent variable, assuming that the score decreased proportionally with increasing exposure to phthalates after receiving TPN solutions. The average of repeated measures of urinary phthalate metabolites was calculated. Each model was adjusted for only birth weight and length of stay in the NICU, owing to the small sample size and for the levels of individual urinary phthalate metabolite measured at admission (before starting TPN). We applied regression imputation to replace the missing urinary phthalate metabolites for ten preterm neonates for whom we could not obtain urine at admission with the mean of each analyte. β regression coefficients and 95% confidence intervals (95% CIs) were calculated to measure the effect of one unit increase of urinary phthalate metabolites on ASQ-3 scores. For ease of interpretation of the results, β regression coefficients were back-transformed and expressed as a percentage change (Δ%) with 95% CIs using the equation: [exp (β) − 1] × 100. We also exponentiated β regression coefficients to calculate the geometric mean ratios (GMRs) and 95%CIs. The R (programming language) was used for the forest plot.

Analyses were considered statistically significant if *p* < 0.05. We also defined *p* < 0.10 as a marginally significant effect due to our study's small sample size and exploratory nature. IBM SPSS Statistics for Windows, version 25.0 (IBM Corp., Armonk, NY, USA) was used for statistical analysis and data management.

### Ethics approval and consent to participate

Each patient's parent signed an informed consent approved by the King Faisal Specialist Hospital and Research Centre Research Ethics Committee.

## Results

### Characteristics of the study population

Between January 16, 2020, and March 22, 2021, 33 premature neonates were admitted to NICU and enrolled in this study. There were nine sets of twins. Among enrolled preterm neonates, 10 were born with a birthweight < 1500 g, and common morbidities were retinopathy of prematurity (*N* = 13), respiratory distress syndrome (*N* = 21), prolonged rupture of membrane (*N* = 12), and intraventricular hemorrhage (*N* = 5), bronchopulmonary dysplasia (*N* = 2), and necrotizing enterocolitis (*N* = 2). Out of 33 neonates, 6 (18.2%) were delivered vaginally, and 27 (81.8%) by Cesarean section. There were 15 (45.5%) females and 18 (54.5%) males. All preterm neonates received TPN solutions, with an average of 5.2 received, ranging from 2 to 16 days. DEP, BB*z*P, DBP, and DEHP were measured in 195 TPN solutions, and all samples had DEP and BB*z*P above the MDL of 0.05 and 0.047 µg/L, respectively. DBP and DEHP were detected at 25.6% and 82.6% above the MDLs of 0.169 and 0.05 µg/L, respectively.

Table 1 shows the demographics and clinical characteristics of the studied neonates, extracted from the patient's medical chart or measured during their stay.

**Table 1:** The demographic and clinical characteristics of premature neonates admitted to the NICU (2020–2021).

Demographic		*N*	Mean	SD	Median	Min	Max
At admission	Mother’s age (years)	24	32.83	5.670	33.00	19	42
	Gestational age (weeks)	33	30.515	2.623	31.00	24	34
	Birth body weight (kg)	33	1.410	0.331	1.42	0.77	2.19
	Head circumference (cm)	32	28.203	2.177	28.50	21.00	32.00
	Crown heel length (cm)	33	39.318	5.056	40.00	23.00	50.00
	Apgar 1-min score	32	5.719	1.464	6.00	3.00	8.00
	Apgar 5-min score	31	7.677	1.045	8.00	5.00	9.00
	Creatinine in urine (mg/dl)	23	12.048	9.540	8.904	3.581	44.250
During NICU stay							
	Number of TPN solutions given to 33 preterm neonates	230	5.20	3.413	5.00	2	16
	DEP in TNP solutions (µg/L)	195	1.983	0.940	2.019	0.209	6.681
	BBP in TNP solutions (µg/L)	195	8.753	3.738	8.427	1.074	24.952
	DBP in TNP solutions (µg/L)	195	0.646	1.557	0.085	0.085	11.036
	DEHP in TNP solutions (µg/L)	195	0.880	0.675	0.645	0.025	4.232
	Creatinine in urine (mg/dl)	254	11.854	6.669	10.278	2.047	58.918
At discharge	Length of stay (days)	33	41.455	26.011	33.00	10.0	108.0
	Age of infants (months)	33	1.197	0.059	1.187	1.14	1.37
	Bodyweight of infants (kg)	33	2.078	0.336	2.02	1.53	3.04
ASQ-3 screening	Age (months)	30	4.155	0.629	4.173	3.32	5.65
	Age (months)-corrected for prematurity	30	2.002	0.002	2.002	2.00	2.00
	Body weight (kg)	27	5.362	1.269	5.2	2.10	7.3
	Body length (cm)	26	62.538	6.797	61.750	50.00	75.00

Clinical therapy		Yes/no *N* (%)					
Antenatal steroids for lung maturation	33	30 (90.9%)/3 (9.1%)					
Antenatal magnesium sulfate-neuroprotective	33	21 (63.6%)/12 (36.4%)					
Maternal chorioamnionitis	33	3 (9.1%)/30 (90.9%)					
Mechanical ventilation	33	27 (81.8%)/6 (18.2%)					
Received blood transfusion	33	9 (27.3%)/24 (72.7%)					

Neonatal comorbidity		Yes/no *N* (%)					
Respiratory distress syndrome	33	21 (63.6%)/12 (36.4%)					
Head US-Intraventricular hemorrhage	33	5 (15.2%)/28 (84.8%)					
Prolonged rupture of membrane	33	12 (36.4%)/21 (63.6%)					
Retinopathy of prematurity	33	13 (39.4%)/20 (60.6%)					
Bronchopulmonary dysplasia	33	2 (6.06)/31 (93.9%)					
Necrotizing enterocolitis	33	2 (6.06)/31 (93.9%)					

### Phthalates assessment

All urine samples (collected at admission and before discharge) contained M*n*BP and MEHP above the MDLs of 1.758 and 2.072 µg/L, respectively. MEP, M*i*BP, MB*z*P, MECPP, MEHHP, and MEOHP were 99.6%, 97.5%, 70%, 99.3%, 90.1%, and 92.6% above the MDLs of 1.597, 1.508, 0.986, 1.569, 1.619, and 0.605 µg/L, respectively. The ratio of samples (before: after TPN) higher than the MDL were 0:1 (MEP), 20:56 (M*i*BP), 11:187 (MB*z*P), 21:260 (MECPP), 16:239 (MEHHP), and 14:248 (MEOHP).

Table 2 presents the urinary levels of MEP, M*i*BP, M*n*BP, MB*z*P, MECPP, MEHHP, MEOHP, and MEHP in preterm neonates at admission and before discharge. The GEE models showed that among the tested phthalates, the means of MB*z*P (χ^2^ = 6.85, *p* = 0.009), MECPP (χ^2^ = 36.781, *p* < 0.001), MEHHP (χ^2^ = 23.033, *p* < 0.001), MEOHP (χ^2^ = 30.634, *p* < 0.001), MEHP (χ^2^ = 9.791, *p* = 0.002), ∑_4_DEHP (χ^2^ = 36.939, *p* < 0.001), and ∑_3_DEHP (χ^2^ = 33.822, *p* < 0.001) in the preterm neonates differed significantly at admission and before discharge. Post hoc analysis with a Bonferroni adjustment revealed that preterm neonates before discharge had a significant increase in urinary levels of MB*z*P [2.024 µg/L (95%CI 1.194, 3.432)], MECPP [12.207 µg/L (95%CI 5.436, 27.413)], MEHHP [7.33 µg/L (95%CI 3.251, 16.544)], MEOHP [11.201 µg/L (95%CI 4.764, 26.364)], MEHP [1.672 µg/L (95%CI 1.212, 2.309)], ∑_4_DEHP [6.449 µmol/L (95%CI 3.536, 11.763)], and ∑_3_DEHP [9.767 µmol/L (95%CI 4.531, 21.031)]. The ICC for serial measurements of urinary phthalate metabolites and the molar sum of the metabolites were moderate (< 0.75) for M*n*BP (0.697) and MEHP (0.612) and excellent (> 0.75) for MEP (0.811), M*i*BP (0.759), MB*z*P (0.874), MECPP (0.912), MEHHP (0.867), MEOHP (0.856), ∑_4_DEHP (0.892), ∑_3_DEHP (0.890), and ∑DBP (0.828).

**Table 2:** Descriptive statistics of urinary phthalate metabolites (µg/L and µg/g creat) in premature neonates (admission and before discharge from the NICU).

µg/L/µmol/L*		MEP	M*i*BP	M*n*BP	MB*z*P	MECPP	MEHHP	MEOHP	MEHP	∑_4_DEHP*^a^	∑_3_DEHP*^b^	∑DBP*^c^
Admission	*N*	23	23	23	23	23	23	23	23	23	23	23
	Mean	43.324	70.070	66.652	5.520	102.004	19.604	15.445	27.279	0.548	0.450	0.615
	SD	53.350	256.421	101.772	6.701	281.192	27.668	19.218	24.336	1.050	1.032	1.254
	Median	20.771	17.719	31.891	0.493	23.282	3.438	2.042	20.082	0.231	0.146	0.216
	GM	21.165	13.157	37.231	1.972	21.285	6.068	3.162	21.513	0.260	0.137	0.271
	Minimum	1.715	0.754	9.817	0.493	0.785	0.810	0.303	7.260	0.045	0.006	0.048
	Maximum	210.817	1245.260	482.075	18.822	1344.489	115.005	55.188	118.737	4.994	4.938	5.963
Before discharge	*N*	260	260	260	260	260	260	260	260	260	260	260
	Mean	43.145	43.882	44.466	10.254	2366.259	180.544	142.531	87.192	9.089	8.775	0.398
	SD	57.804	172.155	40.728	17.730	7297.163	385.587	332.675	394.359	26.147	25.963	0.818
	Median	28.318	19.825	34.116	5.952	191.036	58.325	48.234	30.103	1.304	1.116	0.249
	GM	28.339	18.532	35.315	3.991	259.821	44.494	35.431	35.982	1.675	1.336	0.262
	Minimum	0.849	0.754	7.833	0.493	3.044	0.810	0.303	6.974	0.080	0.015	0.076
	Maximum	541.513	2035.631	269.273	213.769	70,715.085	3267.002	2648.245	5614.190	249.092	247.455	9.559
χ^2^		1.6	1.08	0.076	6.85	36.781	23.033	30.634	9.791	36.939	33.882	0.021
*p*-value		0.206	0.299	0.783	0.009	< 0.001	< 0.001	< 0.001	0.002	< 0.001	< 0.001	0.885

µg/g creat/µmol/g creat**		MEP	M*i*BP	M*n*BP	MB*z*P	MECPP	MEHHP	MEOHP	MEHP	∑_4_DEHP**^d^	∑_3_DEHP**^e^	∑DBP**^f^
Admission	*N*	23	23	23	23	23	23	23	23	23	23	23
	Mean	397.146	385.377	769.925	55.821	948.317	181.389	151.872	268.039	5.174	4.211	3.466
	SD	460.426	979.242	1351.862	74.774	2882.969	272.776	215.181	183.868	10.593	10.555	8.813
	Median	205.271	172.171	310.181	10.826	220.458	36.091	20.182	214.101	2.648	1.609	1.549
	GM	213.586	132.772	375.721	19.897	214.799	61.236	31.913	217.097	2.622	1.381	1.130
	Minimum	19.383	1.704	65.904	1.568	8.875	9.091	2.577	42.443	0.531	0.072	0.008
	Maximum	2089.714	4835.962	6432.809	278.925	14,005.096	1197.967	755.786	875.637	52.022	51.435	43.520
Before discharge	***N	254	254	254	254	254	254	254	254	254	254	254
	Mean	413.910	479.221	461.981	112.477	26,347.736	1916.795	1498.423	1068.453	100.930	97.091	4.312
	SD	503.326	2008.225	461.041	283.930	86,008.559	4227.952	3657.460	5584.138	306.658	304.720	18.073
	Median	282.407	188.541	331.228	56.408	1861.139	577.441	469.884	306.902	13.754	11.652	1.697
	GM	273.666	177.009	337.929	38.382	2451.587	424.373	336.250	347.286	15.901	12.628	1.582
	Minimum	2.891	3.449	50.636	1.334	26.135	2.191	1.030	32.874	0.402	0.112	0.016
	Maximum	5289.247	26,327.353	3844.993	4091.267	901,475.366	41,349.900	37,255.952	74,055.079	3204.346	3191.664	236.927
χ^2^		1.324	0.768	0.229	4.906	34.489	21.017	28.79	5.943	30.699	30.985	0.961
*p*-value		0.25	0.381	0.632	0.027	< 0.001	< 0.001	< 0.001	0.015	< 0.001	< 0.001	0.327

SD: standard deviation; GM: geometric mean; *µmol/L; **µmol/g creat; ***four urine samples with no creatinine reading.

^a^∑_4_DEHP calculated according to the following formula: Sum (∑) MEHP (µg/l)/MWT_MEHP_ (278.34 g/mol) + MEHHP (µg/l)/MWT _MEHHP_ (294.34 g/mol) + MEOHP (µg/l)/ MWT _MEOHP_ (293.33 g/mol) + MECPP (µg/l)/MWT _MECCP_ (308.33 g/mol).

^b^∑_3_DEHP calculated according to the following formula: Sum (∑) MEHHP (µg/l)/MWT _MEHHP_ (294.34 g/mol) + MEOHP (µg/l)/MWT _MEOHP_ (293.33 g/mol) + MECPP (µg/l)/MWT _MECCP_ (308.33 g/mol).

^c^∑DBP calculated according to the following formula: Sum (∑) M*i*BP (µg/l)/MWT_M*i*BP_ (222.24 g/mol) + M*n*BP (µg/l)/MWT _M*n*BP_ (222.24 g/mol).

^d^∑_4_DEHP calculated according to the following formula: Sum (∑) MEHP (µg/g creat)/MWT_MEHP_ (278.34 g/mol) + MEHHP (µg/g creat)/MWT _MEHHP_ (294.34 g/mol) + MEOHP (µg/g creat)/ MWT _MEOHP_ (293.33 g/mol) + MECPP (µg/g creat)/MWT _MECCP_ (308.33 g/mol).

^e^∑_3_DEHP calculated according to the following formula: Sum (∑) MEHHP (µg/g creat)/MWT _MEHHP_ (294.34 g/mol) + MEOHP (µg/g creat)/ MWT _MEOHP_ (293.33 g/mol) + MECPP (µg/g creat)/MWT _MECCP_ (308.33 g/mol).

^f^∑DBP calculated according to the following formula: Sum (∑) M*i*BP (µg/g creat)/MWT_M*i*BP_ (222.24 g/mol) + M*n*BP (µg/g creat)/MWT _M*n*BP_ (222.24 g/mol).

Similar results were obtained when urinary phthalate and molar sum metabolites were calculated based on µg/g creat and µmol/g creat, respectively.

Table 3 presents the Spearman rank correlations for phthalates in TPN and their urinary metabolites in preterm neonates individually and on a molar sum basis. The only significant correlation was observed between DEP (a parent plasticizer) and its urinary metabolite MEP (*r*_s_ = 0.243, *p* = 0.001). Our results show that correlations between parent compounds and other metabolites are not directly related through the same metabolic pathways. In addition, all the phthalates (except DEP) were inversely correlated with either their metabolites, such as DBP and M*i*BP (*r*_s_ = − 0.164) or M*n*BP (*r*_*s*_ = − 0.219), or other metabolites originating from different parent compounds.

**Table 3:** Spearman ranks correlations between the levels of eight phthalate metabolites (µg/L) in the urine of preterm neonates receiving daily TPN solutions during their stay in the NICU and the levels of phthalate compounds in TPN solutions.

		MEP	M*i*BP	M*n*BP	MB*z*P	MECPP	MEHHP	MEOHP	MEHP	∑_4_DEHP	∑_3_DEHP	∑DBP
DEP	*r*_*s*_	0.243**	0.197**	0.231**	0.305**	− 0.093	0.097	0.081	0.012	− 0.033	− 0.039	0.218**
	*p*	0.001	0.010	0.002	< 0.001	0.225	0.207	0.290	0.875	0.667	0.609	0.004
DBP	*r*_*s*_	− 0.212**	− 0.164*	− 0.219**	− 0.262**	0.068	− 0.105	− 0.089	0.122	0.045	0.004	− 0.203**
	*p*	0.005	0.031	0.004	0.001	0.371	0.168	0.247	0.109	0.557	0.962	0.007
BB*z*P	*r*_*s*_	− 0.312**	− 0.236**	− 0.287**	− 0.100	0.113	0.003	− 0.062	0.176*	0.096	0.081	− 0.299**
	*p*	< 0.001	0.002	< 0.001	0.190	0.137	0.964	0.417	0.021	0.211	0.287	< 0.001
DEHP	*r*_*s*_	− 0.296**	− 0.235**	− 0.264**	− 0.359**	0.097	− 0.121	− 0.094	− 0.007	0.018	0.028	− 0.271**
	*p*	< 0.001	0.002	< 0.001	< 0.001	0.206	0.112	0.218	0.923	0.817	0.714	< 0.001

**Correlation is significant at the 0.01 level (2-tailed); *Correlation is significant at the 0.05 level (2-tailed).

Most phthalate metabolites were significantly correlated to various degrees with one another (Table S8). Metabolites from the same parent compound, particularly DEHP, were strongly correlated, with the highest correlation observed between MEOHP and MEHHP (*r*_*s*_ = 0.957). A similar pattern was observed between DBP metabolites (M*i*BP and M*n*BP), with a correlation of 0.579.

### Identification of risk factors associated with levels of phthalates

We looked at the relationship between various cofounders and the levels of phthalate metabolites in urine samples collected from preterm neonates. Inverse correlations were seen between body weight and MECPP (*r*_*s*_ = − 0.554, *p* = 0.001), MEHHP (*r*_*s*_ = − 0.403, *p* = 0.02), MEOHP (*r*_*s*_ = − 0.352, *p* = 0.045), ∑_4_DEHP (*r*_*s*_ = − 0.504, *p* = 0.003), and ∑_3_DEHP (*r*_*s*_ = − 0.516, *p* = 0.002). The same metabolites were inversely correlated with head circumference. No gender differences were seen in the levels of phthalates.

We used Spearman rank correlations to examine the relationship between the length of stay in the NICU and body weight before discharge with urinary phthalate metabolites measured in samples collected from the preterm neonates before discharge. Positive relationships were seen between the length of stay in the NICU and MECPP (*r*_*s*_ = 0.392, *p* = 0.024), ∑_4_DEHP (*r*_*s*_ = 0.346, *p* = 0.049), and ∑_3_DEHP (*r*_*s*_ = 0.349, *p* = 0.046). Results are presented in Table 4. Similar results were obtained when urinary phthalate and molar sum metabolites were calculated based on µg/g creat and µmol/g creat, respectively.

**Table 4:** Bivariate analyses between the levels of phthalate metabolites in urine collected from preterm neonates at NICU and some confounding variables.

Admission		Phthalate metabolites in urine (µg/L)										
		MEP	M*i*BP	M*n*BP	MB*z*P	MECPP	MEHHP	MEOHP	MEHP	∑_4_DEHP	∑_3_DEHP	∑DBP
Mother age (years) ^a^	*r*_*s*_	0.273	− 0.054	− 0.168	0.160	− 0.053	0.001	− 0.027	− 0.123	− 0.071	− 0.058	− 0.077
	*p*	0.124	0.764	0.350	0.373	0.771	0.996	0.881	0.494	0.696	0.750	0.670
	*N*	33	33	33	33	33	33	33	33	33	33	33
Gestational age ^a^	*r*_*s*_	− 0.074	0.027	0.085	0.183	− 0.342	− 0.247	− 0.213	− 0.250	− 0.293	− 0.298	− 0.020
	*p*	0.682	0.883	0.638	0.307	0.052	0.166	0.234	0.161	0.097	0.092	0.913
	*N*	33	33	33	33	33	33	33	33	33	33	33
Birth body weight (Kg) ^a^	*r*_*s*_	0.050	0.058	0.076	0.232	− 0.554**	− 0.403*	− 0.352*	− 0.156	− 0.504**	− 0.516**	0.029
	*p*	0.783	0.748	0.675	0.194	0.001	0.020	0.045	0.385	0.003	0.002	0.872
	*N*	33	33	33	33	33	33	33	33	33	33	33
Head circumference (cm) ^a^	*r*_*s*_	0.010	0.099	0.196	0.235	− 0.453**	− 0.422*	− 0.384*	− 0.134	− 0.438*	− 0.447*	0.156
	*p*	0.958	0.591	0.282	0.196	0.009	0.016	0.030	0.464	0.012	0.010	0.393
	*N*	32	32	32	32	32	32	32	32	32	32	32
Crown heel length (cm) ^a^	*r*_*s*_	− 0.053	− 0.050	0.037	0.089	− 0.336	− 0.251	− 0.278	0.023	− 0.295	− 0.297	− 0.035
	*p*	0.770	0.782	0.837	0.622	0.056	0.159	0.117	0.898	0.096	0.094	0.845
	*N*	33	33	33	33	33	33	33	33	33	33	33
Apgar 1-min score ^a^	*r*_*s*_	0.336	0.122	0.019	0.024	− 0.007	− 0.011	0.055	− 0.289	− 0.018	− 0.016	0.027
	*p*	0.060	0.504	0.917	0.898	0.971	0.953	0.765	0.109	0.924	0.929	0.882
	*N*	32	32	32	32	32	32	32	32	32	32	32
Apgar 5-min score ^a^	*r*_*s*_	0.233	− 0.040	− 0.094	− 0.066	0.278	0.153	0.230	− 0.172	0.228	0.238	− 0.100
	*p*	0.207	0.832	0.615	0.722	0.130	0.412	0.212	0.356	0.218	0.198	0.592
	*N*	31	31	31	31	31	31	31	31	31	31	31
Gender ^b^		0.420	0.619	0.107	0.300	0.877	0.414	0.277	0.495	0.321	0.402	0.154

Before discharge		Phthalate metabolites in urine										
		MEP	M*i*BP	M*n*BP	MB*z*P	MECPP	MEHHP	MEOHP	MEHP	∑_4_DEHP	∑_3_DEHP	∑DBP
Body weight (Kg) ^a^	*r*_*s*_	− 0.062	− 0.084	− 0.146	− 0.241	0.179	0.071	0.034	0.161	0.154	0.144	− 0.098
	*P*	0.733	0.641	0.418	0.177	0.320	0.694	0.849	0.370	0.391	0.425	0.588
	*N*	33	33	33	33	33	33	33	33	33	33	33
Length of stay in the NICU ^a^	*r*_*s*_	0.055	− 0.049	0.013	− 0.246	0.375*	0.273	0.263	0.145	0.346*	0.349*	0.070
	*p*	0.762	0.787	0.941	0.167	0.032	0.124	0.139	0.419	0.049	0.047	0.697
	*N*	33	33	33	33	33	33	33	33	33	33	33
Blood transfusion ^b^		0.069	0.442	0.052	0.374	0.312	0.777	0.628	0.686	0.396	0.396	0.082

^a^Spearman rank correlation test [**Correlation is significant at the 0.01 level (2-tailed)/ *Correlation is significant at the 0.05 level (2-tailed)].

^b^Mann-Whitney test.

### Neurodevelopment assessment

Table 5 shows that 1, 4, and 1 infants had scores lower than the cutoff of 22.77 (communication), 41.84 (gross motor), and 30.16 (fine motor), respectively. All exhibited low scores in only one domain. We also tried cutoffs based on 1SD/2SD below the mean of each domain. Out of the 30 infants, 80% scored below 1SD, with one infant scoring low in three developmental domains (gross motor, problem-solving, and personal-social) and 7 in two of the five domains. A score below 2SD was seen in 16.7% of infants, with only one scoring low in two domains (gross motor and problem-solving). Among the five domains, problem-solving scores were significantly and positively correlated with birth weight (*r*_s_ = 0.396, *p* = 0.03) and crown-heel length (*r*_*s*_ = 0.47, *p* = 0.009). Positive relationships were observed between the mother's age and gross motor (*r*_s_ = 0.405, *p* = 0.026) and personal-social scores (*r*_s_ = 0.389, *p* = 0.034). None of the five domains showed differences between males and females or was associated with the length of stay in the NICU (*p* > 0.05).

**Table 5:** Assessment of ASQ-3 at the age of 2 months old.

ASQ3-5 domains	*N*	Mean	SD	Percentiles			Min	Max	ASQ-3		Below the mean		*N* (%) below the mean	
									Cutoff	*N* (%)				
											1SD	2SD	1SD	2SD
				25th	50th	75th								
Communication	30	45.000	10.828	40.00	45.00	55.00	20.00	60.00	22.77	1 (3.3)	34.17	23.34	5 (16.7)	1 (3.3)
Gross motor	30	55.167	8.146	50.00	60.00	60.00	30.00	60.00	41.84	4 (13.3)	47.02	38.88	4 (13.3)	1 (3.3)
Fine motor	30	51.667	9.223	43.75	55.00	60.00	30.00	60.00	30.16	1 (3.3)	42.44	33.22	7 (23.3)	1 (3.3)
Problem-solving	30	55.167	8.039	50.00	60.00	60.00	30.00	60.00	24.62	None	47.13	39.09	3 (10)	2 (6.7)
Personal social	30	50.833	5.584	50.00	50.00	55.00	40.00	60.00	33.71	None	45.25	39.67	5 (16.7)	None

*Below cutoff, the infant needs further assessment with a professional; **Close to the cutoff means the infant needs monitoring.

Bivariate analysis was conducted to test neonatal comorbidies listed in Table 1 with a sufficient number of observations (≥ 5). Only preterm neonates with retinopathy of prematurity had lower personal-solving scores, which was marginally significant (*p* = 0.08).

Inverse relationships were observed between DEHP in TPN solutions and personal-social scores (*r*_s_ = − 0.433, *p* = 0.035). Surprisingly, a moderate positive correlation was seen between DEP and fine motor scores (*r*_s_ = 0.764, *p* < 0.001). Results are shown in Table S9.

### Crude and multiple regression models

**Figure 2:**
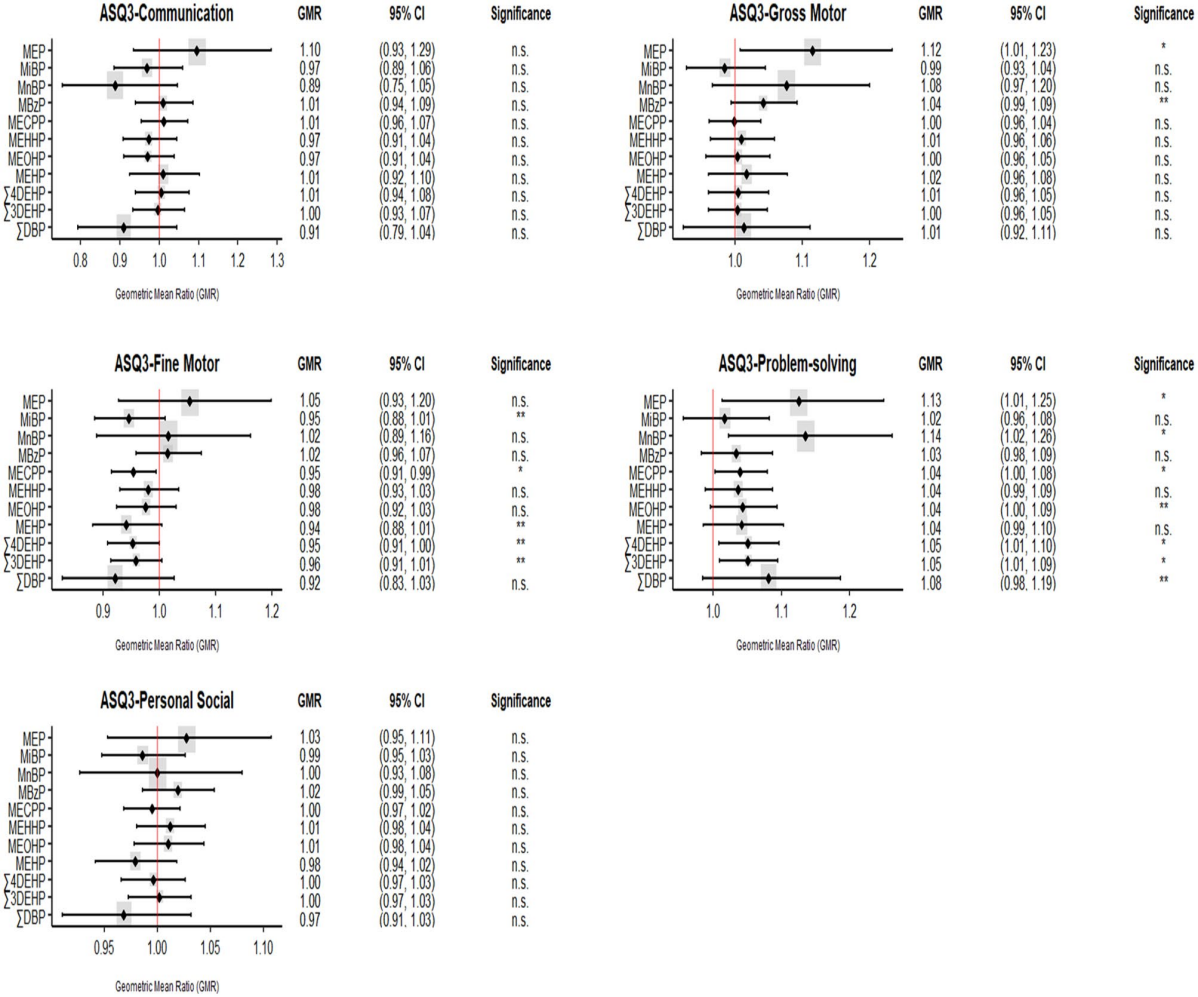
Forest plot showing adjusted associations between scores of the five ASQ-3 domains and urinary phthalate metabolites. The estimated effects presented as geometric mean ratio (GMR) and 95% confidence intervals (95% CIs). The vertical red line represent no effect and the horizontal lines represent the 95%CI. Confidence intervals that do not cross the red vertical line are significant at *p < 0.05 or **p < 0.1; n.s.: not significant.

We used linear regression models to evaluate the crude and adjusted effects of phthalate exposure after receiving TPN solutions on infants' neurodevelopmental performance at 2 months. As shown in Table 6, a decrease in ASQ-3 fine motor scores was associated with elevated urinary levels of M*i*BP (Δ% = − 5.4, 95%CI − 11.6, 1), MECPP (Δ% = − 4.6, 95%CI = − 8.5, − 0.6), MEHP (Δ% = − 5.9, 95%CI = − 11.8, 0.5), ∑_4_DEHP (Δ% = − 4.7, 95%CI = − 9.2, 0), and ∑_3_DEHP (Δ% = − 4.1, 95%CI = − 8.6, 0.5) in preterm neonates before discharge. An increase in ASQ-3 problem-solving scores in preterm neonates was associated with higher urinary MEP (Δ% = 12.5, 95%CI 1.3, 25.0), M*n*BP (Δ% = 13.5, 95%CI 2.2, 26.2), MECPP (Δ% = 4.0, 95%CI 0.3, 7.9), ∑_4_DEHP (Δ% = 5.1, 95%CI 0.8, 9.6) and ∑_3_DEHP (Δ% = 5.1, 95%CI 0.9, 9.4). Elevated levels of urinary MEP was associated with 11.5% increase (95%CI 0.8, 25.0) in gross motor scores. All models were adjusted for birth weight and the length of stay in the NICU and respective urinary phthalate metabolite measured at admission. Our findings were also clearly visualized using forest plot displaying based on adjusted GMRs and 95% CIs between ASQ-3 scores and urinary phthalate metabolites (Fig. 2).

**Table 6:** The adjusted relationship between ln-transformed ASQ-3 scores of infants at the age of 2-month and urinary phthalate metabolites (µg/g creat) and molar sums of the metabolites (µmol/g creat) measured before their discharge from the NICU.

Analyte	Δ% (95%CI)				
	Communication	Gross motor	Fine motor	Problem-solving	Personal social
MEP	9.6 (− 6.6, 28.5)	11.5 (0.8, 23.4)*	5.4 (− 7.3, 20.0)	12.5 (1.3, 25.0)*	2.7 (− 4.7, 10.7)
M*i*BP	− 3.1 (− 11.5, 6.0)	− 1.5 (− 7.2, 4.5)	− 5.4 (− 11.6, 1.0)**	1.7 (− 4.4, 8.2)	− 1.4 (− 5.3, 2.6)
			− 5.8 (− 12.0, 0.8)**^a^		
			− 5.0 (− 11.1, 1.5)^b^		
			− 5.5 (− 11.8, 1.1)**^c^		
			− 5.7 (− 12.1, 1.2)**^d^		
M*n*BP	− 11.2 (− 24.6, 4.6)	7.7 (− 3.3, 20.0)	1.6 (− 11.1, 16.2)	13.5 (2.2, 26.2)*	0.0 (− 7.3, 8.0)
MB*z*P	1.0 (− 6.0, 8.5)	4.2 (− 0.6, 9.2)**	1.5 (− 4.2, 7.5)	3.4 (− 1.7, 8.7)	1.9 (− 1.4, 5.3)
MECPP	1.2 (− 4.5, 7.3)	− 0.1 (− 3.8, 3.9)	− 4.6 (− 8.5, − 0.6)*	4.0 (0.3, 7.9)*	− 0.5 (− 3.1, 2.1)
			− 4.5 (− 8.4, − 0.4)*^a^		
			− 4.7 (− 8.5, − 0.7)*^b^		
			− 4.9 (− 9.2, − 0.4)*^c^		
			− 4.6 (− 8.6, − 0.5)*^d^		
MEHHP	− 2.7 (− 9.2, 4.4)	1.0 (− 3.6, 5.9)	− 2.0 (− 7.0, 3.5)	3.7 (− 1.2, 8.7)	1.2 (− 2.0, 4.5)
MEOHP	− 2.9 (− 9.1, 3.9)	0.4 (− 4.3, 5.2)	− 2.4 (− 7.6, 3.0)	4.3 (− 0.4, 9.3)**	1.0 (− 2.2, 4.4)
MEHP	1.0 (− 7.5, 10.2)	1.7 (− 3.9, 7.8)	− 5.9 (− 11.8, 0.5)**	4.2 (− 1.5, 10.3)	− 2.1 (− 5.8, 1.8)
			− 6.3 (− 12.4, 0.2)**^a^		
			− 6.4 (− 12.2, − 0.2)*^b^		
			− 5.9 (− 12.1, 0.7)**^c^		
			− 6.4 (− 12.7, 0.5)**^d^		
∑_4_DEHP	0.5 (− 6.1, 7.6)	0.5 (− 3.9, 5.0)	− 4.7 (− 9.2, 0.0)**	5.1 (0.8, 9.6)*	− 0.4 (− 3.4, 2.6)
			− 4.6 (− 9.2, 0.2)**^a^		
			− 4.7 (− 9.2, − 0.1)*^b^		
			− 5.0 (− 9.9, 0.3)**^c^		
			− 4.7 (− 9.3, 0.2)**^d^		
∑_4_DEHP	− 0.3 (− 6.7, 6.5)	0.4 (− 3.9, 4.8)	− 4.1 (− 8.6, 0.5)**	5.1 (0.9, 9.4)*	0.2 (− 2.8, 3.1)
			− 4.0 (− 8.5, 0.7)**^a^		
			− 4.0 (− 8.3, 0.5)**^b^		
			− 4.3 (− 9.2, 0.7)**^c^		
			− 4.1 (− 8.7, 0.6)**^d^		
∑DBP	− 9.0 (− 20.7, 4.5)	1.3 (− 7.7, 11.2)	− 7.9 (− 17.3, 2.6)	8.1 (− 1.6, 18.6)	− 3.1 (− 9.0, 3.1)

Data also expressed as percent change (Δ%) and their corresponding 95% confidence intervals (CI) in score for every one unit increase in the metabolite. Bold characters denoted significant associations.

*p* < 0.05; **p < 0.1

Each linear regression model was adjusted for phthalate metabolite levels in urine taken at admission, birth body weight and length of stay in the NICU.

Regression model was further adjusted for ^a^respiratory distress syndrome; ^b^intraventricular hemorrhage; ^c^premature prolonged rapture of the membrane; ^d^retinopathy of prematurity.

The crude results were roughly similar, although some became either marginally significant or non-significant after adjusting for confounders, such as the association between communication scores and urinary M*n*BP levels (Table S10).

Further, multiple regression analyses were performed only for urinary phthalate metabolites that exhibited negative association with ASQ-3 fine motor scores after adjusting separately for neonatal comorbidities such as respiratory distress syndrome, intraventricular hemorrhage, prolonged rupture of membrane and retinopathy of prematurity. The results remained the same except the association between M*i*BP and ASQ-3 fine motor scores became non-significant after adjusting for intraventricular hemorrhage.

## Discussion

### Main findings of the study

This prospective study was the first to assess the impact of exposure to phthalates through TPN solutions on the neurodevelopment status of 30 preterm neonates when they reached the age of 2 months. Urinary levels of MB*z*P, MECPP, MEHHP, MEOHP, MEHP, ∑_4_DEHP, and ∑_3_DEHP increased significantly in preterm neonates before discharge from the NICU. The daily dose of MEP, DBP, BB*z*P, and DEHP for our preterm neonates measured from their TPN solutions was much lower than the recommended tolerable daily limit. However, these levels were not correlated with their phthalate parent compounds in the TPN solutions, suggesting other sources of exposure in the NICU, such as devices, tubings, or staff/family members. Urinary DEHP metabolites were associated with reduced head circumference and birth weight. After adjusting for gestational age, the inverse relationships remained significant, suggesting the direct effects of phthalates. Finally, exposure of preterm neonates to DEHP during their stay in the NICU was associated with neurodevelopment scores in infants at the age of 2 months, mainly in fine motor skills. We also observed that increased levels of some phthalate metabolites were positively associated with problem-solving and gross motor skills; although this observation is unexpected, it was reported previously^42–44^.

### Exposure of preterm neonates to phthalates during their NICU stay via TPN and its potential association with low neurodevelopment scores at the age of 2 months

We observed a 2- to 23-fold increase in the urinary excretion of MB*z*P, MECPP, MEHHP, MEOHP, and MEHP in preterm neonates before discharge. Yet, there was no association between these metabolites and their parenteral phthalates (BB*z*P and DEHP) in TPN solutions, suggesting that TPN might not be the source of exposure in our study. TPN bags used for our preterm neonate were labeled free of DEHP, and phthalate traces were detected in 83% of the TPN solutions, ranging between 0.379 to 4.232 µg/L, reflecting contamination during preparation. The maximum daily intake was 0.272 µg/kg body weight; this is much lower than the European Food Safety Authorities (EFSA) tolerable daily intake (TDI) limit of 50 μg/kg body weight/day^45^ or the Environmental Protection Agency (EPA) oral reference dose (RfD) of 20 μg/kg body weight/day^46^. It has been reported that depending on the lipid content added in TPN solutions, infants and children who receive long-term TPN might be regularly exposed to non-negligible amounts of DEHP ranging from 80 to 200 µg per day^47^. Despite the low content of DEHP in TPN solutions, our preterm neonates had been significantly exposed to DEHP, as shown by the high levels of its four metabolites (MECPP, MEHHP, MEOHP, and MEHP). These levels increased with the length of stay of the preterm neonates in the NICU. Unlike adults, in whom phthalates tend to have a shorter half-life (5–24 h)^48^, phthalates in preterm neonates are likely to have a longer half-life owing to the 'neonates' immature hepatic and renal clearance^49,50^. Despite the half-life of phthalates being short enough that they do not persist in the body, one should consider the adverse health effects from repeated, occasional, and long-term exposure. Short and long-term health risks are related to phthalate exposure in NICU, mainly subfertility, bronchopulmonary dysplasia, necrotizing enterocolitis, parenteral nutrition-associated cholestasis, and neurodevelopmental disorders^51^.

Few researchers have assessed DEHP exposure in NICU patients based on a single metabolite^52^ or either multiple ones or their sum^19,53–55^. Although our median values of MECPP (191.036 µg/L), MEHHP (58.325 µg/L), MEOHP (48.234 µg/L), and MEHP (30.103 µg/L) were ≥ fourfold higher than Stroustrup et al.^19^, they were 5–6 times lower than those observed by Su et al.^55^ and Weuve et al.^53^, apart from MEHP which was lower in those studies than our value. Demirel et al.^23^ noted that high excretion of MEHHP in the first urine sample of preterm neonates with birth weight < 1000 g might be associated with renal immaturity.

Nearly 80% of our preterm neonates who received TPN had detectable MB*z*P in their urine with a maximum concentration of 213.769 µg/L, and its parent compound (BB*z*P) was found in all solutions at concentrations between 1.075 and 24.952 µg/L. Vinyl flooring in the NICU might represent a hidden source of BB*z*P exposure to our preterm neonates^53,56^. The highest dose received by one preterm neonate was 3.226 µg/kg body weight/day, which is much lower than the EFSA's TDI of 500 µg/kg body weight/day^57^ or the EPA's RfD of 200 µg/kg body weight/day^58^. Our median MB*z*P value was almost 5–7 times lower than those reported by Stroustrup et al.^19^ and Weuve et al.^53^ at 27.37 and 41 µg/L, respectively.

DEP and ∑DBP are usually used as fixatives in perfumes, cosmetics, and personal care products^59,60^. All our preterm neonates had detectable MEP (a metabolite of DEP) and M*n*BP (a metabolite of D*n*BP) in urine, and 98.8% had M*i*BP (a metabolite of D*i*BP). Our median values of MEP (28.31 µg/L), M*i*BP (19.825 µg/L), and M*n*BP (34.116 µg/L) in preterm neonates were comparable to the values of 21.13, 15.26, and 36.82 µg/L, respectively, reported by Stroustrup et al.^42^, but higher than Weuve et al.^53^ for M*n*BP (18 µg/L). The highest MEP, M*i*BP, and M*n*BP were 541.51, 2035.63, and 268.28 µg/L, respectively. Whereas DEP was detected in all TPN solutions, ranging from 0.209 to 6.68 µg/L, only 20% of the solutions had DBP between 0.026 and 11.04 µg/L. Our data also demonstrated that DEP in TPN solution was significantly associated with urinary MEP levels in our preterm neonates, leading to a daily dose between 0.023 and 0.751 µg/kg body weight. None of our preterm neonates received a daily DEP dose exceeding the RfD of 800 μg/kg body weight^61^, suggesting that TPN might not be a significant source of exposure in our patients. The contribution of DBP in TPN to our preterm neonates' daily dose ranged between 0.002 and 1.427 µg/kg body weight, much lower than the RfD of 100 µg/kg body weight^62^.

Our data were 3-, 4-, 14-, 7-, 9-, and 21-fold higher for M*n*BP, M*i*BP, MECPP, MEHHP, MEOHP, and MEHP, respectively, than values reported by national surveys such as the National Health and Nutrition Examination Survey in the USA (CDC, 2019). Our findings suggest that phthalates in TPN solutions do not seem to significantly contribute to the high urinary levels of metabolites in preterm neonates. However, DEHP metabolites tended to increase with an extended stay in the NICU. Other possible sources, such as contact with NICU staff or family members who may have applied fragranced personal care products, received medications or blood transfusion, or been in contact with medications, devices, or cleaning materials may be the reason for their high urinary metabolite levels^17,19^.

In line with other studies, we observed negative associations between phthalate metabolites in the urine of preterm neonates and some birth anthropometric measures^63,64^. In our study population, smaller head circumference and low birth weight were associated with increased urinary levels of MECPP, MEHHP, MEOHP, ∑_4_DEHP, and ∑_3_DEHP. Because anthropometric measurements are expected to influence preterm delivery^65^, we reexamined these relationships after adjusting for gestational age. Head circumference and birth weight remained significantly and inversely associated only with MECPP, ∑_4_DEHP, and ∑_3_DEHP (*p* < 0.01). Although our study did not measure phthalate metabolites in the cord or maternal blood, phthalates can cross the placenta to the fetus, with a significant correlation between metabolites in the maternal blood and the cord^66^. In general, phthalates have a short half-life that can reflect the source of exposure in the preceding 1 or 2 days^48^. Because urine was collected from our preterm neonates immediately after birth, we believe that phthalate metabolites may originate from the mother and be transmitted. It is possible to assume that maternal exposure to phthalates, particularly DEHP, was associated with preterm delivery seen in the population, as reported by others^67^.

The implications of altered birth anthropometric parameters such as head circumference and birth weight in preterm neonates on child neurodevelopment have been reported^68–70^. Furthermore, exposure to phthalates, particularly DEHP, has been linked with neurodevelopment^71^, although data are still limited and inconsistent. As we reported earlier, exposure of our preterm neonates to phthalates, mostly DEHP, increased significantly at the end of their NICU stay. In this study, we observed inverse associations between fine motor scores and elevated levels of urinary M*i*BP, MECPP, MEHP, ∑_4_DEHP, and ∑_3_DEHP. Studies have shown that neonatal comorbidity such as respiratory distress, intraventricular hemorrhage, prolonged rupture of membrane, bronchopulmonary dysplasia, and necrotizing enterocolitis can impact neurodevelopmental outcomes^72–74^. The relationship between lower scores in fine motor and some urinary phthalate metabolites remained significant even after controlling for some neonatal comorbidity that we obtained. The risk of neurodevelopmental delay increases as the gestational age at birth decreases^75,76^. The World Health Organization classified preterm neonates into three categories based on gestational age: extremely preterm (< 28 weeks), very preterm (28–32 weeks), and moderate to late preterm (32 to 37 weeks)^77^. Since the gestational age of our studied preterm neonates falls within the three categories, we reexamined the inverse association between fine motor scores and M*i*BP, MECPP, MEHP, ∑_4_DEHP, and ∑_3_DEHP after controlling the regression models for gestational age. The results remained the same, where increases in all phthalate metabolites (except for M*i*BP) were associated with 3.8 to 6.5% decreases in fine motor scores. Because small head circumference predicts abnormal brain volume associated with neurodevelopment delays^78,79^, we adjusted our regression model for head circumference. Similar results were obtained, and a significant decrease of − 4.9 (95% CI − 8.5, − 1.1), − 5.8 (− 11.8, 0.5), − 5.3 (95%CI − 9.6, − 0.8), and − 4.6 (− 8.7, − 0.3) in fine motor scores was observed with elevated MECPP, MEHP, ∑_4_DEHP, and ∑_3_DEHP, respectively. For M*i*BP, there was a decrease in fine motor scores of − 5.1 (95% CI − 11.1, 1.4) but not significant.

Despite the link between head circumference, intracranial volume, and brain size, its association with cognitive function remains unclear because of risk factors' direct and indirect effects (nutritional, socioeconomic, environmental, and other anthropometric measures)^80^. For example, a study found the association between prenatal manganese and neurodevelopment scores was mediated by birth length rather than head circumference^81^. Our results may reflect a complex interplay between phthalate exposure and anthropometric measures that need to be explored to understand better the relationship between exposure and neurodevelopment in infants born prematurely. Impaired fine motor skills may hinder several aspects of daily functions ranging from basics, such as grasp strength, to more complex visual-motor integration that affects learning and writing abilities, subsequently influencing academic performance^82^. Exposure to endocrine disruptors such as DEHP can alter thyroid hormones critical for brain development^83,84^, particularly the cerebellum, associated with fine motor coordination^85^. Preterm neonates are also at high risk of impaired fine motor skills^86^. Surprisingly, we observed that increased urinary levels of some phthalate metabolites in preterm neonates were associated with better problem-solving and gross motor scores. A similar finding was observed between ∑DEHP and attention/social performance in preterm neonates^42^. The authors suggested that exposure to phthalates may follow a non-linear trajectory that accelerates the development of specific neural networks. Other researchers related improved behavioral development during the first years of life and prenatal exposure to certain phthalates to gender that might have a modifier role^43,44^. Although the relationship between maternal exposure to phthalates and neurodevelopment in children has also been examined in human studies^71^, only one study assessed preterm neonates during their NICU stay Stroustrup et al.^42^. In general, studies showed an inconsistent pattern in the association between exposure to phthalates and neurodevelopmental outcomes that was related to various factors such as exposure misclassification, critical window of exposure, sex-specific effects, and the effects of phthalate mixtures^87,88^.

Lastly, we should mention that the non-significant findings in our study might result from low statistical power owing to the small sample size, and additional research is needed using the data of a larger cohort.

### Study limitations, strengths, and future direction

The present study's limitations should be acknowledged. First, the small sample size may have limited the statistical power to detect the detrimental effect of certain phthalates. The reasons for not achieving our targeted sample size (*N* = 150) were partially the outbreak of the SARS-CoV-2 virus and also because some preterm neonates did not require TPN solutions. However, we tried to optimize our study's statistical power by choosing continuous outcome variables rather than dichotomized outcome variables in the regression analyses. This is a valid approach used in epidemiological studies with small samples^89,90^. Furthermore, a recent study recommended a sample size of ≥ 25 for fixed-effect regression models^91^. Second, selection bias, including only high-risk preterm neonates, could have affected the results. Third, we tested specific neonatal comorbidities associated with neurodevelopment delay, particularly intraventricular hemorrhage^92^. No significant relationship was seen because only five preterm neonates were affected, which undermined the validity of intraventricular hemorrhage as a risk factor. Fourth, several potential confounders that might affect exposure and scores, such as genetic susceptibility and socioeconomic and nutritional status, were not considered. Fifth, there was a lack of adjustment in the statistical analysis for risk factors that impact preterm neonates' neurodevelopment, such as glucose and sodium abnormalities^93^ and metabolic complications related to parenteral nutrition^94,95^. Sixth, the number of measurements for each patient varied, either because sampling the urine was difficult or because the NICU nurse missed reserving it for the study after usage (TPN units). Seventh, 2 months may be too early to estimate infant neurodevelopment. Eighth, the ASQ-3 has higher sensitivity and specificity in older infants and children^96^. Ninth, the ASQ-3 is a questionnaire-based assessment for parents that reflects their negative or positive views, and misunderstandings of questions may lead to underestimating or overestimating an infant's developmental scores^97^. Tenth, residual confounding from unmeasured factors may have led to unexpected associations. Last, co-exposure to other pollutants could also affect the neurodevelopmental outcomes of our infants.

Nonetheless, the study has some strengths, which include (1) its prospective design; (2) exposure assessments being taken at two points (admission and before discharge); (3) daily measurements of phthalate metabolites in the urine of the preterm neonates receiving TPN solutions; (4) daily measurements of four phthalate compounds in TPN solutions received by the preterm neonates during their stay in the NICU; and (5) neurodevelopment assessments at 2 and 18 months (ongoing).

Our current research also examines whether the association between early exposure to phthalates in the NICU and neurodevelopment persists in our infants at the age of 18 months, using a range of neurocognitive tests to ensure an in-depth evaluation of various neurodevelopmental aspects, including autism.

## Conclusions

This prospective study is the first to assess the impact of exposure to phthalates through TPN solutions on the neurodevelopment status of 30 preterm neonates at the age of 2 months. Although our sample size was small, our analyses revealed some potentially relevant findings. Urinary levels of MB*z*P, MECPP, MEHHP, MEOHP, MEHP, ∑_4_DEHP, and ∑_3_DEHP increased significantly in preterm neonates before discharge from the NICU, which was not associated with receiving TPN solutions but might have come from other sources. Elevated urinary MECPP, MEHHP, MEOHP, and MEHP metabolites in our preterm neonates indicate intensive use of DEHP devices/materials in the NICU. At 2 months, exposure of preterm neonates to DEHP in the NICU was associated with neurodevelopment scores in infants, mainly in fine motor skills. Some phthalate metabolites were positively associated with gross motor and problem-solving skills. Given the extreme vulnerability of our population, it is critical to minimize their exposure to phthalates during their NICU stay.

## Supplementary Information

Supplementary Information.

## Data Availability

The data that support the findings of this study are available from King Faisal Specialist Hospital and Research Centre, but restrictions apply to the availability of these data, which were used under license for the current study, and so are not publicly available. Data are, however, available from the authors upon reasonable request and with permission of King Faisal Specialist Hospital and Research Centre.
