# Supplementary material for: Exposure of preterm neonates receiving total parenteral nutrition to phthalates and its impact on neurodevelopment at the age of 2 months
Source: Sci Rep. 2023 Apr 28;13:6969. doi: 10.1038/s41598-023-33715-w (PMC10141929; doi:10.1038/s41598-023-33715-w)
Supplement: Supplementary file 1 — Supplementary Information. [file 41598_2023_33715_MOESM1_ESM.docx]

**Exposure of preterm neonates receiving total parenteral nutrition to phthalates and its impact on neurodevelopment at the age of 2 months**

**Iman Al-Saleh^a^**, Rola Elkhatib**^a^**, Hissah Alnuwaysir^a^, Hesham Aldhalaan^b^, Eiman Alismail^b^, Abdulaziz Binmanee^c^, Amal Hawari^c^, [Fahad Alhazzani](http://www.kfshrc.edu.sa/en/home/person/47970)^c^, [Mohammad Bin Jabr](http://www.kfshrc.edu.sa/en/home/person/84808)^c^, Gamal Mohamed^d^

^a^ Environmental Health Program, ^b^ Center for Autism Research, ^c^ Neonatal Critical Care Section, Department of Pediatrics, ^d^ Biostatistics, Epidemiology and Scientific Computing Department, King Faisal Specialist Hospital and Research Centre, P.O. Box: 3354, Riyadh 11211, Saudi Arabia

**^a^ Corresponding Address:**

Dr. Iman Al-Saleh (MBC#03)

Environmental Health Program

King Faisal Specialist Hospital & Research Centre

P.O. Box: 3354, Riyadh 11211, Saudi Arabia

E-mail: [iman@kfshrc.edu.sa](mailto:iman@kfshrc.edu.sa)/

1. Analysis of urinary phthalate metabolites

The method used in this study was adopted and modified from previously published studies (Servaes et al., 2013; Silva et al., 2003; Silva et al., 2007; Wang et al., 2013).

- 1. Chemicals

Eight phthalate metabolites standards: monoethyl phthalate (MEP), mono-*n*-butyl phthalate (M*n*BP), mono-iso-butyl phthalate (M*i*BP), monobenzyl phthalate (MB*z*P), mono-(2-ethylhexyl) phthalate (MEHP), mono-(2-ethyl-5-hydroxyhexyl) phthalate (MEHHP), mono-(2-ethyl-5-oxohexyl) phthalate (MEOHP), and mono-(2-ethyl-5-carboxypentyl) phthalate (MECPP) and their isotopically labeled internal standards (IS), MEP ^13^C_4_, M*n*BP ^13^C_4_, MB*z*P ^13^C_4_, MEHP ^13^C_4_, MEHHP ^13^C_4_, MEOHP ^13^C_4_ and MECPP ^13^C_4_ were purchased from Cambridge Isotope Laboratories, Inc. (Andover, MA, USA). These compounds were obtained as solutions (100 µg/mL) in methyl tertiary-butyl ether (MTBE) with 95% chemical purity. A 200 U/mL of the enzyme β-Glucuronidase (*Escherichia coli*-K12) and 4-methylumbelliferyl (4-MeUmb) glucuronide were purchased from Roche Biomedical (Mannheim, Germany). A 100 µg/mL of 4-methylumbelliferone (4-MeUmb) and it's isotopically labelled IS 4-methylumbelliferone ^13^C_4_ (4-MeUmb ^13^C_4_) solutions in acetonitrile (99% purity) were also obtained from Cambridge Isotope Laboratories, Inc. (Andover, MA, USA). The following chemicals (their manufacturers) were used in this method: acetonitrile and sodium phosphate dibasic (Fisher Scientific); acetic acid, phosphoric acid, and potassium phosphate monobasic (Sigma Aldrich Laboratories, Inc. St. Louis, MO, USA); Ammonium acetate (BDH Chemical Ltd Poole, England); and formic acid (Fluka Analytical). All the glassware was rinsed with methanol and dried before use.

- 1. Instrument

Ultra-Performance Liquid Chromatography (UPLC)-tandem MS (LC-MS/MS) was purchased from Waters (Milford, MA, USA). The ACQUITY UPLC H-Class System consisted of an integrated solvent manager, sample manager, and column heating configured for quaternary solvent delivery and sample introduction via a direct inject. The UPLC system was linked to a Waters Xevo^®^ TQ-S Micro tandem quadrupole mass spectrometer using a negative electrospray ionization interface (ESI). Argon was used as the collision gas at a flow of 0.22 mL/min. The mass spectrometer parameters were as follows: electrospray source block and desolvation temperature 150 °C and 500 °C, respectively; cone and desolvation nitrogen gas flow 1 l/h and 1000 l/h, respectively; and a capillary voltage of 0.54 kV. The cone voltage and collision energy were compound-dependent by direct infusion of the compound into the mass spectrometer. Both parameters were optimized to obtain maximum sensitivity with the highest amount of product ions available. Out of these results, characteristic precursor and product ions were selected for detection in the multiple reaction monitoring (MRM) mode. The two highest precursor/product ion transitions were chosen for each compound, as listed in **Table S2**. Instrument control, data acquisition, and processing were performed using MassLynx V 4.1 software (Waters, Milford, MA, USA). Each ion of interest in the chromatogram was selected and integrated automatically by the program.

The phthalate metabolites were separated on an ACQUITY UPLC BEH phenyl column (100 mm × 2.1 mm; 1.7 µm). The column temperature was maintained at 40 °C. The optimum separation was obtained with a binary mobile phase constituted of ultrapure water (solvent A) and acetonitrile (solvent B), both solvents acidified with 0.1% acetic acid. For solvent A, the gradient program was as follows: 0 min (77%), 1.64 min (75%), 4.35 min (67%), 7.5 min (55%), 10.0 min (100%), 10.01 min (77%) and 12 min (77%). For solvent B, the gradient was as follows: 0 min (23%), 1.64 min (25%), 4.35 min (33%), 7.5 min (45%), 10.0 min (100%), 10.1 min (23%), and 12 min (23%).

- 1. Sample preparation

Frozen urine samples were thawed, vortexed, and sonicated for 5 min. An aliquot of 200 µL of the urine sample was spiked with 50 µL of 1 µg/mL IS solution containing seven isotopically labeled phthalate metabolites and 4-Mu ^13^C_4_ at a concentration of 0.05 µg/mL and 4-MeUmb glucuronide at a concentration of 0.15 μg/mL. The sample was then buffered with 250 µl of an ammonium acetate buffer solution (pH 6.5) containing 10 µL of a β-glucuronidase solution and incubated at 37°C for 90 minutes to hydrolyze the analyte conjugates. The addition of 4-MeUmb glucuronide to the urine sample was done to monitor the enzymatic hydrolytic efficiency exerted by β-glucuronidase (Blount et al., 2000b). After enzymatic de-conjugation, the samples were acidified with phosphate buffer (pH 2) and loaded onto an OASIS HLB SPE glass cartridge (Waters, Milford, MA, USA) (5cc/200 mg), which was preconditioned with 2 ml of acetonitrile and 2 ml phosphate buffer (pH 2). After extraction using a Visiprep™ Vacuum Manifold system (Supleco, Bellefonte, PA, USA), the cartridge was then washed with 2 mL of 0.1 M formic acid and 4 ml of UPLC water. The phthalates were eluted with 2 ml acetonitrile and evaporated to dryness. After the drying, the residue was re-suspended in 200 µl of UPLC water and transferred to a glass auto-sampler vial insert for analysis. The injection volume was 10 µl. The results were presented as micrograms per liter (µg/L).

- 1. Method validation

The analytical method was validated using blank (or spiked) urine samples to demonstrate linearity, method detection limit (MDL), accuracy, and precision (**Table S3**). The calibration curves were evaluated with matrix-matched calibration standards in urine samples in the range of 5-200 µg/l. All calibration curves gave good linearity, with R^2^ >0.9999. MDLs in urine samples were determined according to the United States Environmental Protection Agency procedures (US EPA, 2012) by analyzing ten replicate urine samples. Each replicate must be processed through the entire analytical method. The MDL was calculated as follows: MDL=*t*_(n−1, α=0.01)_×s, (*n:* the number of replicate analyses, *s:* the standard deviation (SD) of the replicate analyses, and *t:* the Student's *t* value for *n* − 1 degree of freedom at 99% confidence level). To check the method's precision, we analyzed ten replicates of urine samples spiked with three concentrations of the intermediate spiking solutions (15, 80, and 150 µg/L) on different days (day-to-day precisions) and within the same day (within-day precisions) for all compounds studied including 4-MeUmb under the same optimized experimental conditions. The analytical recoveries and precisions obtained for the three spiked levels are listed in **Table S4**. Most of the recoveries for these chemicals were above 95%. Precision values, expressed as relative SDs (RSD %), were lower than 15% for all phthalates. The results demonstrated an acceptable precision.

- 1. External quality control

Urine samples from G-EQUAS (German External Quality Assessment Scheme, Germany: <http://www.g-equas.de/>) proficiency tests organized by the German University of Erlangen-Nuremberg (Institute and Out-Patient Clinic for Occupational, Social and Environmental Medicine) were used to check the validity of the method. Two Certified Reference samples were received from G-EQUAS proficiency tests (rounds 56 and 63) containing either a low (sample reference 9A) or a high (sample reference 9B) of M*i*BP, M*n*BP, MECPP, MEOHP, MB*z*P, MEHHP, and MEHP. They were analyzed in duplicate at the beginning and end of each run. Mean results for all runs and the G-EQUAS tolerance ranges are presented in [**Table**](http://www.sciencedirect.com/science/article/pii/S157002321400004X#tbl0025) **S5**. Z-score was calculated to measure the deviation of our findings from the actual reference value. As shown in **Table S6**, most results were satisfactory (z ≤ 2), with a few exceptions. MEHHP and MEOHP (Reference A and B for both) were lower than the expected range with large deviations. Langlois et al. (2012) related bias in urinary phthalate metabolites to the neat standards used for calibration curve preparation. The authors notably pinpointed those purchased from Cambridge Isotope Laboratories (the one used in this study); they tested and found variations in reporting M*n*BP for different rounds of G-EQUAS. As we know, G-EQUAS established its reference values based on the consensus values of participating laboratories instead of reference laboratories.

The z-score for our in-house spiked urine samples with three different phthalate metabolites levels was calculated and revealed satisfactory for all compounds with a z-score ≤ 2, as shown in **Table S7**. This indicates the suitability of our method for the determination of urinary phthalate metabolites.

1. Analysis of phthalate compounds in TPN:

*2.1. Chemicals and materials.* Organic HPLC-grade solvents, including methanol and acetone, were purchased from Fischer Scientific (Pittsburgh, PA, USA), but dichloromethane was supplied from Merck (Darmstadt, Germany). Diethyl phthalate (DEP), dibutyl phthalate (DBP), benzyl butyl phthalate (BBP), and diethylhexyl phthalate (DEHP) were obtained as neat compounds with purities of 99.5% from Chemservice (USA). Deuterated di-*n*-propylphthalate-3,4,5,6-d_4_ (DPrP-d_4_) was used as an internal standard (IS) and was obtained from Fluka Chemie GmbH (Steinheim, Germany). For SPME, 65 μm polydimethylsiloxane-divinylbenzene (PDMS/DVB) fibers were purchased from Bellefonte, PA, USA.

*2.2. Analytical instrumentation*. The gas chromatography/mass spectrometer (GC/MS) was composed of a 6890N gas chromatograph and a 5973 quadrupole mass-selective spectrometer (Agilent Technologies, Palo Alto, CA, USA). Data acquisition and analysis were performed with Agilent Chemstation software (G1701 DJ version). The column was an Agilent J&W DB-5MS capillary column (cross-linked poly 5% diphenyl, 95% dimethylsiloxane), 30 m × 0.25 mm (i.d.) × 0.25 μm film thickness. An Agilent inlet liner (4 mm i.d.) packed with glass wool was used for the injector. The GC/MS was equipped with a CombiPAL autosampler that has a 32-sample tray, a cooler tray, a SPME fiber holder, a temperature controller and a 20 mL-vial agitator. The PDMS/DVB fiber (65 μm) was selected on the recommendations of Carrillo et al. (2008) and Liu (2008) The fiber was conditioned for 1 h at 250 °C following the manufacturer's instructions. Each fiber was used for approximately 40 injections.

The GC oven temperature was programmed from 80 °C (held for 0.5 min) to 220 °C at 10 °C min^−1^ and was heated to 290 °C at 30 °C min^−1^ (held for 4 min) (total analysis time = 20.83 min). The MS was auto-tuned with perfluorotributylamine (PFTBA, tuning standard). Electron ionization (EI) was performed at 70 eV. The ion source temperature was set to 250 °C, and the helium carrier gas flow rate (purity 99.999%) was set to 1 ml/min. The temperature of the ion source was maintained at 250 °C. The splitless mode (held for 2 min) was used for the injections, the split flow was set to 50 mL/min, and the injector temperature was maintained at 300 °C. The MS system was set to selected ion monitoring (SIM) mode with a solvent delay of 8 min. The SIM mode was also used for quantitative determination. Three fragment ions were monitored for each compound. The fragments were selected after injecting standard solutions in full-scan mode and determining their retention times. The most characteristic ion in the spectrum was selected for quantification, and the other two ions were selected for confirmation, as displayed in **Table S8**. The areas of the peak were used for quantitation. The reported values of phthalates were presented as µg/L.

*2.3.Sample analysis.* Each 5 mL of TPN sample was diluted with up to 10 mL of deionized water. For each SPME analysis, 10 mL of the diluted sample spiked with 10 μL of IS (2 µg/L) was placed into 20 ml glass vial~~s~~ (75.5 mm long and 22.5 mm diameter). The final concentration of IS in the sample was 0.002 µg/L. Then, the vial was tightly capped with a 1.5 mm~~,~~ PTFE/silicone septum. The samples were mixed well and then left to equilibrate for 10 min at 40 ^o^C. The sample vial was then moved to the CombiPAL autosampler agitator. SPME was conducted at 90 °C for 13 min at an agitation rate of 500 rpm. The analytes were thermally desorbed from the SPME fiber into the GC/MS inlet at 270 °C for 5 min. The optimum extraction time for most of the phthalates was 30 min. The vial penetration depth was set to 25 mm, and the tip of the SPME fiber was 1.0 cm above the surface of the sample solution. Three replicates were measured for each TPN sample.

*2.4.Standard solutions:* Individual stock solutions of DEP, DBP, BBP and DEHP (1000 µg/mL) were prepared in 10 mL dichloromethane. Appropriate aliquots of individual stock solutions were diluted with methanol to make a mixture of an intermediate spiking solution at a concentration of 2 µg/mL each, from which calibration standards were in the range of 0.5-16 μg/L (DEP, DBP, BBP and DEHP). Stock and spiking standards were stored in the dark at 4 °C until use. TPN solutions are liquid samples, which primarily consist of water. Therefore, calibration standards were prepared in deionized water. A blank standard, which only contained deionized water and the IS, was also included. The six phthalates and the IS were analyzed in less than 21 min.

All of the calibration curves showed good linearity, with *r*^2^ in the range of 0.997 to 0.999. The precision of the method was evaluated within the same day (within-run precision) and on different days (between-run precision) at three concentration levels of 1.5, 3.0, and 6 µg/L for DEP, DBP, BBP, and DEHP. The results are presented in **Table S9**. The RSDs were lower than 10% for all phthalates. The recoveries were satisfactory in the range between 89% and 101%.

*3.* Creatinine (creat) in urine was estimated using a commercial Creatinine ELISA Kit from Oxford Biomedical Research (Oxford, MI). The absorbance (OD_450_) was measured using a microplate reader from BioTek Instruments, Winooski, VT, USA. Levels I and II of the Lyphochek urine quality-control sample (Bio-Rad Laboratories, Irvine, CA) for 16 runs were 71.93 ± 4.82 mg/dL and 213.14 ± 9.27 mg/dL, respectively. Both fell within the range of the certified values for Level I: 64.2-96.2 mg/dL and Level II: 190-285 mg/dL with RSD < 7%.

**Table S1**: STROBE Statement—Checklist of items that included in the current study

|  | Item No | Recommendation | Section |
| --- | --- | --- | --- |
| **Title and abstract** | 1 | (*a*) Indicate the study’s design with a commonly used term in the title or the abstract | Abstract |
|  |  | (*b*) Provide in the abstract an informative and balanced summary of what was done and what was found | Abstract |
| Introduction | | |  |
| Background/rationale | 2 | Explain the scientific background and rationale for the investigation being reported | Paragraphs 1-3 |
| Objectives | 3 | State specific objectives, including any prespecified hypotheses | Paragraph 4 |
| Methods | | |  |
| Study design | 4 | Present key elements of study design early in the paper | Section 2.1 |
| Setting | 5 | Describe the setting, locations, and relevant dates, including periods of recruitment, exposure, follow-up, and data collection | Section 2.1 |
| Participants | 6 | (*a*) Give the eligibility criteria, and the sources and methods of selection of participants. Describe methods of follow-up | Section 2.1 |
|  |  | (*b*) For matched studies, give matching criteria and number of exposed and unexposed | N/A |
| Variables | 7 | Clearly define all outcomes, exposures, predictors, potential confounders, and effect modifiers. Give diagnostic criteria, if applicable | Section 2.6 |
| Data sources/ measurement | 8* | For each variable of interest, give sources of data and details of methods of assessment (measurement). Describe comparability of assessment methods if there is more than one group | Sections 2.4 and 2.5 |
| Bias | 9 | Describe any efforts to address potential sources of bias | Section 2.6 |
| Study size | 10 | Explain how the study size was arrived at | N/A |
| Quantitative variables | 11 | Explain how quantitative variables were handled in the analyses. If applicable, describe which groupings were chosen and why | Sections 2.4 and 2.5 |
| Statistical methods | 12 | (*a*) Describe all statistical methods, including those used to control for confounding | Section 2.6 |
|  |  | (*b*) Describe any methods used to examine subgroups and interactions | N/A |
|  |  | (*c*) Explain how missing data were addressed | Section 2.6 |
|  |  | (*d*) If applicable, explain how loss to follow-up was addressed | N/A |
|  |  | (*e*) Describe any sensitivity analyses | N/A |
| Results | | |  |
| Participants | 13* | (a) Report numbers of individuals at each stage of study—eg numbers potentially eligible, examined for eligibility, confirmed eligible, included in the study, completing follow-up, and analysed | Sections 2.1 and 2.5 and Table 1. |
|  |  | (b) Give reasons for non-participation at each stage | Section 4.3 |
|  |  | (c) Consider use of a flow diagram | Figure 1 |
| Descriptive data | 14* | (a) Give characteristics of study participants (eg demographic, clinical, social) and information on exposures and potential confounders | Table 1 |
|  |  | (b) Indicate number of participants with missing data for each variable of interest | Table 1 |
|  |  | (c) Summarise follow-up time (eg, average and total amount) | N/A |
| Outcome data | 15* | Report numbers of outcome events or summary measures over time | Section 3.1 |
| Main results | 16 | (*a*) Give unadjusted estimates and, if applicable, confounder-adjusted estimates and their precision (eg, 95% confidence interval). Make clear which confounders were adjusted for and why they were included | Sections 2.6, 3.5, and Table 6. |
|  |  | (*b*) Report category boundaries when continuous variables were categorized | N/A |
|  |  | (*c*) If relevant, consider translating estimates of relative risk into absolute risk for a meaningful time period | N/A |
| Other analyses | 17 | Report other analyses done—eg analyses of subgroups and interactions, and sensitivity analyses | N/A |
| Discussion | | |  |
| Key results | 18 | Summarise key results with reference to study objectives | Section 4.1 |
| Limitations | 19 | Discuss limitations of the study, taking into account sources of potential bias or imprecision. Discuss both direction and magnitude of any potential bias | Section 4.3 |
| Interpretation | 20 | Give a cautious overall interpretation of results considering objectives, limitations, multiplicity of analyses, results from similar studies, and other relevant evidence | Last paragraph of section 4.2/study limitations |
| Generalisability | 21 | Discuss the generalisability (external validity) of the study results | N/A |
| Other information | | |  |
| Funding | 22 | Give the source of funding and the role of the funders for the present study and, if applicable, for the original study on which the present article is based | Acknowledgment |

*Give information separately for exposed and unexposed groups.

**Note:** An Explanation and Elaboration article discusses each checklist item and gives methodological background and published examples of transparent reporting. The STROBE checklist is best used in conjunction with this article (freely available on the Web sites of PLoS Medicine at http://www.plosmedicine.org/, Annals of Internal Medicine at http://www.annals.org/, and Epidemiology at http://www.epidem.com/). Information on the STROBE Initiative is available at http://www.strobe-statement.org.

**Table S2:** Monitored ions of the target analytes and collision energies used for detection.

| **Compounds** | **Retention Time (min)** | **Precursor ion** | **Quantifier ion (m/z)** | **Qualifier ion (m/z)** | **Collision energy (eV)** | **Cone Voltage (V)** |
| --- | --- | --- | --- | --- | --- | --- |
| 4-MeUmb | 1.42 | 174.89 | 118.82 | 146.85 | 22 | 56 |
| MEP | 1.71 | 192.96 | 76.81 | 120.83 | 14 | 26 |
| M*i*BP | 4.14 | 221.048 | 70.88 | 76.93 | 12 | 2 |
| M*n*BP | 4.34 | 221.048 | 76.87 | 71.00 | 18 | 28 |
| MECPP | 4.64 | 307.0319 | 120.79 | 158.95 | 24 | 2 |
| MB*z*P | 5.14 | 255.095 | 76.97 | 104.54 | 22 | 30 |
| MEHHP | 4.45 | 292.92 | 120.88 | 145.00 | 18 | 2 |
| MEOHP | 4.68 | 291.098 | 76.92 | 142.97 | 34 | 2 |
| MEHP | 8.77 | 276.86 | 133.93 | 127.028 | 14 | 6 |
| **IS** |  |  |  |  |  |  |
| ^13^C_4_-4-MeUmb | 1.42 | 178.89 | 120.88 |  | 22 | 18 |
| ^13^C_4_-MEP | 1.71 | 196.96 | 78.82 |  | 14 | 28 |
| ^13^C_4_-M*n*BP | 4.34 | 225.048 | 78.88 |  | 16 | 24 |
| ^13^C_4_-MECPP | 4.64 | 311.096 | 123.78 |  | 24 | 2 |
| ^13^C_4_-MB*z*P | 5.14 | 259.07 | 106.99 |  | 14 | 28 |
| ^13^C_4_-MEHHP | 4.45 | 297.05 | 144.99 |  | 10 | 2 |
| ^13^C_4_-MEOHP | 4.68 | 295.03 | 123.92 |  | 16 | 2 |
| ^13^C_4_-MEHP | 8.77 | 281.81 | 136.95 |  | 12 | 2 |

**Table S3:** Method validation parameters: regression coefficients (R^2^), method detection limit (MDL), within-day precision, day-to-day precision, recovery (%) of the spiked urine sample with eight phthalate metabolites and 4-MeUmb.

| **Compounds** | **^*^R^2^ ± SD**  ***N*=6** | **MDL (µg/L)**  ***N*=10** | **Within-day (RSD %) *N*=10** | | | **Day-to-day (RSD %)**  ***N*=6** | | | **Recovery (%)**  ***N*=6** | | |
| --- | --- | --- | --- | --- | --- | --- | --- | --- | --- | --- | --- |
|  |  |  | **15 µg/l** | **80 µg/l** | **150 µg/l** | **15 µg/l** | **80 µg/l** | **150 µg/l** | **15 µg/l** | **80 µg/l** | **150 µg/l** |
| 4-MeUmb | 0.99973 ± 0.0002 | 1.901 | 4.5% | 3.4% | 3.7% | 16.3% | 11.6% | 5.7% | 100.1% | 95.4% | 98.9% |
| MEP | 0.99933 ± 0.0005 | 1.697 | 4.0% | 4.7% | 3.4% | 4.5% | 4.5% | 6.4% | 93.3% | 94.4% | 98.5% |
| M*i*BP | 0.99848 ± 0.0014 | 1.508 | 3.8% | 3.1% | 3.0%^**^ | 8.2% | 6.5% | 5.4% | 96.4% | 99.4% | 95.4% |
| M*n*BP | 0.99922 ± 0.0005 | 1.758 | 4.5% | 3.6% | 3.7% | 6.8% | 7.2% | 5.7% | 102.0% | 96.6% | 94.4% |
| MB*z*P | 0.99963 ± 0.0006 | 0.986 | 2.6% | 3.1% | 1.9% | 4.7% | 4.5% | 2.3% | 103.0% | 96.9% | 94.8% |
| MECPP | 0.99898 ± 0.0012 | 1.569 | 3.6% | 4.6% | 3.9% | 6.4% | 5.0% | 6.6% | 99.0% | 95.4% | 96.6% |
| MEHHP | 0.99962 ± 0.0003 | 1.619 | 4.0% | 4.1% | 3.8% | 6.9% | 6.3% | 5.5% | 98.0% | 95.9% | 98.3% |
| MEOHP | 0.99973 ± 0.0003 | 0.605 | 1.5% | 3.2% | 2.1% | 2.1% | 5.4% | 4.9% | 101.4% | 99.0% | 99.0% |
| MEHP | 0.99882 ± 0.0010 | 2.074 | 5.0% | 4.3% | 1.9% | 5.1% | 4.6% | 2.6% | 99.4% | 103.1% | 101.4% |

^*^Results for six runs; ^**^ 9 readings only

**Table S4**: Mean concentration of 7 phthalate metabolites in low and high urine samples from an inter-laboratory test (G-EQUAS 63) and their expected tolerance ranges. Shaded cells present a high unsatisfactory z-score.

| **Compounds** | **Control 56** | **Mean^*^** | **Reference value** | **Expected tolerance range** | **z-score** |
| --- | --- | --- | --- | --- | --- |
| M*i*BP | 9 A | 31.26 | 27.7 | 20.5 <-> 34.9 | 1.117 |
|  | 9 B | 94.47 | 93.0 | 76.2 <-> 109.8 | 0.210 |
| M*n*BP | 9 A | 33.06 | 25.2 | 15.9 <-> 34.5 | 1.869 |
|  | 9 B | 108.77 | 108.0 | 78.6 <-> 137.4 | 0.092 |
| MB*z*P | 9 A | 3.28 | 3.7 | 2.5 <-> 4.9 | -1.002 |
|  | 9 B | 10.47 | 10.4 | 8.0 <-> 12.8 | 0.039 |
| MECPP | 9 A | 28.76 | 25.64 | 18.86 <-> 32.42 | 0.675 |
|  | 9 B | 90.67 | 73.04 | 55.07 <-> 91.01 | 1.938 |
| MEHHP | 9 A | 13.97 | 23.05 | 16.75 <-> 29.35 | -4.466 |
|  | 9 B | 43.32 | 61.75 | 49.21 <-> 74.29 | -3.385 |
| MEOHP | 9 A | 13.69 | 17.81 | 14.06 <-> 21.56 | -4.112 |
|  | 9 B | 44.11 | 54.87 | 47.64 <-> 62.10 | -3.066 |
| MEHP | 9 A | 3.77 | 3.75 | 2.70 <-> 4.80 | 0.034 |
|  | 9 B | 10.02 | 11.06 | 8.69 <-> 13.43 | -0.633 |

**^*^** The mean of four separate runs.

| **Compounds** | **Control 63** | **Mean^*^** | **Reference value** | **Expected tolerance range** | **z-score** |
| --- | --- | --- | --- | --- | --- |
| M*i*BP | 9 A | 15.02 | 13.8 | 10.5 <-> 17.1 | Not calculated because the results represent only two runs. |
|  | 9 B | 42.15 | 49.3 | 38.8 <-> 59.8 |  |
| M*n*BP | 9 A | 9.62 | 7.3 | 5.2 <-> 9.4 |  |
|  | 9 B | 28.30 | 27.8 | 20.3 <-> 35.3 |  |
| MB*z*P | 9 A | 1.94 | 1.5 | 0.9 <-> 2.1 |  |
|  | 9 B | 10.19 | 8.6 | 6.8 <-> 10.4 |  |
| MECPP | 9 A | 3.73 | 4.61 | 3.05 <-> 6.17 |  |
|  | 9 B | 22.40 | 27.74 | 20.66 <-> 34.82 |  |
| MEHHP | 9 A | 4.08 | 3.75 | 2.91 <-> 4.59 |  |
|  | 9 B | 26.77 | 32.96 | 28.01 <-> 37.91 |  |
| MEOHP | 9 A | 3.24 | 2.9 | 2.24 <-> 3.56 |  |
|  | 9 B | 21.33 | 25.5 | 20.97 <-> 30.03 |  |
| MEHP | 9 A | 1.61 | 1.25 | 0.83 <-> 1.67 |  |
|  | 9 B | 7.99 | 8.11 | 6.52 <-> 9.70 |  |

**^*^** The mean of two separate runs.

**Table S5:** Mean, SD, minimum and maximum z-score obtained for six runs of spiked urine samples spiked with three levels of eight phthalate metabolites

| **Urinary phthalate metabolites** | **Concentration (µg/L)** | **Mean** | **SD** | **z-score** |
| --- | --- | --- | --- | --- |
| MEP | 15.0 | 13.997 | 0.630 | -1.590 |
|  | 80.0 | 75.497 | 3.409 | -1.321 |
|  | 150.0 | 147.733 | 9.493 | -0.239 |
| M*i*BP | 15.0 | 14.454 | 1.184 | -0.461 |
|  | 80.0 | 79.521 | 5.156 | -0.093 |
|  | 150.0 | 143.097 | 7.720 | -0.894 |
| M*n*BP | 15.0 | 15.299 | 1.045 | 0.286 |
|  | 80.0 | 77.267 | 5.536 | -0.494 |
|  | 150.0 | 141.625 | 8.100 | -1.034 |
| MB*z*P | 15.0 | 15.443 | 0.722 | 0.613 |
|  | 80.0 | 77.532 | 3.479 | -0.709 |
|  | 150.0 | 142.221 | 3.207 | -2.426 |
| MECPP | 15.0 | 14.854 | 0.950 | -0.153 |
|  | 80.0 | 76.320 | 3.831 | -0.961 |
|  | 150.0 | 144.825 | 9.621 | -0.538 |
| MEHHP | 15.0 | 14.693 | 1.012 | -0.303 |
|  | 80.0 | 76.752 | 4.849 | -0.670 |
|  | 150.0 | 147.393 | 8.096 | -0.322 |
| MEOHP | 15.0 | 15.209 | 0.313 | 0.670 |
|  | 80.0 | 79.172 | 4.307 | -0.192 |
|  | 150.0 | 148.550 | 7.350 | -0.197 |
| MEHP | 15.0 | 14.912 | 0.761 | -0.115 |
|  | 80.0 | 82.503 | 3.783 | 0.662 |
|  | 150.0 | 152.035 | 3.991 | 0.510 |

**Table S6:** The list of tested phthalates, including internal standard (IS), their retention times, quantification and confirmation ions.

| **Phthalate compound** | **Retention time (min)** | **Monitored ions (*m/z*)** | |
| --- | --- | --- | --- |
|  |  | **Quantification** | **Confirmation** |
| DEP | 12.132 | 149 | 105, 177 |
| DBP | 15.993 | 149 | 104, 223 |
| BBP | 18.539 | 149 | 91, 207 |
| DEHP | 19.514 | 149 | 57, 167 |
| DPrP-d_4_ (IS) | 14.216 | 153 | 195, 108 |

**Table S7:** Method validation parameters: regression coefficients (R^2^), method detection limit (MDL), within-day precision, day-to-day precision, recovery (%) of the six phthalate compounds in TPN solutions. Units are in µg/L.

| **Compounds** | **R^2^ ± SD**  ***N*=5** | **MDL (µg/L)**  ***N*=10** | **Within-day (RSD %) *N*=10** | | | **Day-to-day (RSD %)**  ***N*=5** | | | **Recovery (%)**  ***N*=5** | | |
| --- | --- | --- | --- | --- | --- | --- | --- | --- | --- | --- | --- |
|  |  |  | **1.5** | **3.0** | **6.0** | **1.5** | **3.0** | **6.0** | **1.5** | **3.0** | **6.0** |
| DEP | 0.99930 ± 0.00034 | 0.050 | 3.4% | 3.9% | 2.4% | 4.6% | 4.7% | 3.5% | 99.0% | 89.2% | 98.6% |
| BBP | 0.99936 ± 0.00048 | 0.047 | 2.1% | 5.3% | 5.5% | 5.4% | 7.1% | 6.3% | 98.9% | 96.0% | 100.6% |
| DBP | 0.99748 ± 0.00173 | 0.169 | 5.9% | 2.8% | 3.8% | 7.5% | 6.9% | 5.6% | 97.8% | 97.0% | 94.4% |
| DEHP | 0.99782 ± 0.00111 | 0.050 | 4.5% | 5.8% | 6.1% | 7.5% | 7.7% | 4.6% | 97.8% | 90.4% | 95.6% |

**Table S8**: Spearman rank correlations of urinary concentrations of 8 [phthalate](https://www.sciencedirect.com/topics/earth-and-planetary-sciences/phthalates) metabolites in preterm neonates who received TPN during their stay at the NICU.

|  | | **MEP** | **M*i*BP** | **M*n*BP** | **MB*z*P** | **MECPP** | **MEHHP** | **MEOHP** | **MEHP** | **∑_4_DEHP** | **∑_3_DEHP** | **∑DBP** |
| --- | --- | --- | --- | --- | --- | --- | --- | --- | --- | --- | --- | --- |
| **M*i*BP** | *r_s_* | 0.403^**^ |  |  |  |  |  |  |  |  |  |  |
|  | *p* | < 0.001 |  |  |  |  |  |  |  |  |  |  |
|  | *N* | 260 |  |  |  |  |  |  |  |  |  |  |
| **M*n*BP** | *r_s_* | 0.551^**^ | 0.579^**^ |  |  |  |  |  |  |  |  |  |
|  | *p* | < 0.001 | < 0.001 |  |  |  |  |  |  |  |  |  |
|  | *N* | 260 | 260 |  |  |  |  |  |  |  |  |  |
| **MB*z*P** | *r_s_* | 0.260^**^ | 0.388^**^ | 0.391^**^ |  |  |  |  |  |  |  |  |
|  | *p* | < 0.001 | < 0.001 | < 0.001 |  |  |  |  |  |  |  |  |
|  | *N* | 260 | 260 | 260 |  |  |  |  |  |  |  |  |
| **MECPP** | *r_s_* | 0.119 | 0.133^*^ | 0.039 | -0.008 |  |  |  |  |  |  |  |
|  | *p* | 0.055 | 0.032 | 0.536 | 0.894 |  |  |  |  |  |  |  |
|  | *N* | 260 | 260 | 260 | 260 |  |  |  |  |  |  |  |
| **MEHHP** | *r_s_* | 0.193^**^ | 0.373^**^ | 0.210^**^ | 0.406^**^ | 0.823^**^ |  |  |  |  |  |  |
|  | *p* | 0.002 | < 0.001 | 0.001 | < 0.001 | < 0.001 |  |  |  |  |  |  |
|  | *N* | 260 | 260 | 260 | 260 | 260 |  |  |  |  |  |  |
| **MEOHP** | *r_s_* | 0.238^**^ | 0.444^**^ | 0.263^**^ | 0.373^**^ | 0.790^**^ | 0.957^**^ |  |  |  |  |  |
|  | *p* | < 0.001 | < 0.001 | < 0.001 | < 0.001 | < 0.001 | < 0.001 |  |  |  |  |  |
|  | *N* | 260 | 260 | 260 | 260 | 260 | 260 |  |  |  |  |  |
| **MEHP** | *r_s_* | 0.029 | 0.118 | 0.042 | 0.231^**^ | 0.494^**^ | 0.593^**^ | 0.486^**^ |  |  |  |  |
|  | *p* | 0.645 | 0.057 | 0.503 | < 0.001 | < 0.001 | < 0.001 | < 0.001 |  |  |  |  |
|  | *N* | 260 | 260 | 260 | 260 | 260 | 260 | 260 |  |  |  |  |
| **∑_4_DEHP** | *r_s_* | 0.114 | 0.203^**^ | 0.082 | 0.154^*^ | 0.956^**^ | 0.892^**^ | 0.849^**^ | 0.594^**^ |  |  |  |
|  | *p* | 0.067 | 0.001 | 0.188 | 0.013 | < 0.001 | < 0.001 | < 0.001 | < 0.001 |  |  |  |
|  | *N* | 260 | 260 | 260 | 260 | 260 | 260 | 260 | 260 |  |  |  |
| **∑_3_DEHP** | *r_s_* | 0.138^*^ | 0.232^**^ | 0.100 | 0.126^*^ | 0.977^**^ | 0.904^**^ | 0.876^**^ | 0.525^**^ | 0.979^**^ |  |  |
|  | *p* | 0.026 | < 0.001 | 0.108 | 0.042 | < 0.001 | < 0.001 | < 0.001 | < 0.001 | < 0.001 |  |  |
|  | *N* | 260 | 260 | 260 | 260 | 260 | 260 | 260 | 260 | 260 |  |  |
| **∑DBP** | *r_s_* | 0.553^**^ | 0.791^**^ | 0.927^**^ | 0.378^**^ | 0.094 | 0.281^**^ | 0.344^**^ | 0.067 | 0.141^*^ | 0.168^**^ |  |
|  | *p* | < 0.001 | < 0.001 | < 0.001 | < 0.001 | 0.131 | < 0.001 | < 0.001 | 0.282 | 0.023 | 0.007 |  |
|  | *N* | 260 | 260 | 260 | 260 | 260 | 260 | 260 | 260 | 260 | 260 |  |
| ^**^ Correlation is significant at the 0.01 level (2-tailed); ^*^ Correlation is significant at the 0.05 level (2-tailed). | | | | | | | | | | | | |

**Table S9**: Bivariate analyses between the five ASQ-3 domains for preterm infants assessed at two months and some confounding variables.

|  | | **ASQ-3 domains** | | | | |
| --- | --- | --- | --- | --- | --- | --- |
|  |  | **Communication** | **Gross motor** | **Fine motor** | **Problem-solving** | **Personal special** |
| Mother age (years) ^a^ | *r*_s_ | 0.106 | **0.405^*^** | 0.259 | -0.139 | **0.389^*^** |
|  | *P* | 0.578 | **0.026** | 0.168 | 0.464 | **0.034** |
|  | *N* | 30 | **30** | 30 | 30 | **30** |
| Gestational age ^a^ | *r*_s_ | 0.061 | -0.110 | 0.190 | 0.295 | 0.001 |
|  | *p* | 0.751 | 0.565 | 0.314 | 0.113 | 0.997 |
|  | *N* | 30 | 30 | 30 | 30 | 30 |
| Birth weight (Kg) ^a^ | *r*_s_ | 0.235 | 0.084 | 0.282 | **0.396^*^** | 0.190 |
|  | *p* | 0.211 | 0.661 | 0.131 | **0.030** | 0.315 |
|  | *N* | 30 | 30 | 30 | **30** | 30 |
| Head circumference  (cm) ^a^ | *r*_s_ | 0.121 | 0.029 | 0.141 | 0.212 | 0.173 |
|  | *p* | 0.532 | 0.881 | 0.464 | 0.268 | 0.369 |
|  | *N* | 29 | 29 | 29 | 29 | 29 |
| Crown heel length (cm) ^a^ | *r*_s_ | 0.281 | 0.148 | 0.224 | **0.470^**^** | 0.110 |
|  | *p* | 0.132 | 0.434 | 0.235 | **0.009** | 0.562 |
|  | *N* | 30 | 30 | 30 | **30** | 30 |
| Apgar 1-minutes score ^a^ | *r*_s_ | 0.186 | -0.147 | 0.050 | 0.126 | -0.118 |
|  | *p* | 0.335 | 0.446 | 0.798 | 0.515 | 0.541 |
|  | *N* | 29 | 29 | 29 | 29 | 29 |
| Apgar 5-minutes score ^a^ | *r*_s_ | 0.091 | -0.317 | -0.211 | -0.108 | -0.305 |
|  | *p* | 0.646 | 0.100 | 0.282 | 0.583 | 0.115 |
|  | *N* | 28 | 28 | 28 | 28 | 28 |
| Body weight before discharge (Kg) ^a^ | *r*_s_ | -0.031 | 0.209 | -0.338 | -0.211 | -0.004 |
|  | *p* | 0.870 | 0.267 | 0.068 | 0.264 | 0.982 |
|  | *N* | 30 | 30 | 30 | 30 | 30 |
| Length of stay in the NICU (days) | *r*_s_ | -0.212 | 0.109 | -0.326 | -0.328 | -0.128 |
|  | *p* | 0.261 | 0.567 | 0.078 | 0.077 | 0.499 |
|  | *N* | 30 | 30 | 30 | 30 | 30 |

**Table S9-Continued**

|  | | **ASQ-3 domains** | | | | |
| --- | --- | --- | --- | --- | --- | --- |
|  |  | **Communication** | **Gross motor** | **Fine motor** | **Problem-solving** | **Personal special** |
| DEP in TPN (µg/L) ^a^ | *r*_s_ | -0.053 | 0.231 | **0.764^**^** | 0.296 | 0.305 |
|  | *p* | 0.806 | 0.278 | **< 0.001** | 0.161 | 0.147 |
|  | *N* | 24 | 24 | **24** | 24 | 24 |
| DBP in TPN (µg/L) ^a^ | *r*_s_ | 0.115 | -0.329 | -0.276 | 0.150 | -0.312 |
|  | *p* | 0.593 | 0.117 | 0.193 | 0.484 | 0.137 |
|  | *N* | 24 | 24 | 24 | 24 | 24 |
| BBP in TPN (µg/L) ^a^ | *r*_s_ | -0.133 | 0.387 | -0.247 | -0.105 | -0.180 |
|  | *p* | 0.535 | 0.062 | 0.245 | 0.625 | 0.400 |
|  | *N* | 24 | 24 | 24 | 24 | 24 |
| DEHP in TPN (µg/L) ^a^ | *r*_s_ | -0.166 | -0.046 | -0.337 | -0.179 | **-0.433^*^** |
|  | *p* | 0.438 | 0.830 | 0.107 | 0.403 | **0.035** |
|  | *N* | 24 | 24 | 24 | 24 | **24** |
| Gender ^b^ | | 0.482 | 0.881 | 0.568 | 0.789 | 0.416 |
| Respiratory distress syndrome (RDS) ^b^ | | 0.235 | 0.461 | 0.549 | 0.759 | 0.845 |
| Head US-Intraventricular hemorrhage (IVH) ^b^ | | 0.304 | 0.265 | 0.196 | 0.848 | 0.638 |
| Retinopathy of prematurity (ROP) ^b^ | | 0.413 | 0.323 | 0.351 | 0.08 | 0.725 |
| Prolonged rupture of membrane (PROM) ^b^ | | 0,237 | 0.589 | 0.169 | 0.689 | 0.633 |

^a^ Spearman rank correlation test [^**^ Correlation is significant at the 0.01 level (2-tailed)/ ^*^Correlation is significant at the 0.05 level (2-tailed)]; ^b^: Mann-Whitney test

**Table S10**: The crude relationship between ln-transformed ASQ-3 scores of infants at 2 months and urinary phthalate metabolites (µg/g creat) and molar sums of the metabolites (µmol/g creat) measured before their discharge from the NICU. Data expressed as percent change (**Δ%)** and their corresponding 95% confidence intervals (CI) in the score for every one-unit increase in the metabolite. Bold characters denoted significant associations (*p* < 0.05^*^ or *p* < 0.1^**^).

| **Analyte** | **Δ% (95%CI)** | | | | |
| --- | --- | --- | --- | --- | --- |
|  | **Communication** | **Gross motor** | **Fine motor** | **Problem-solving** | **Personal-social** |
| MEP | 7.0 (-10.3, 27.6) | 7.8 (-3.8, 20.8) | 9.2 (-3.9, 24.0) | 6.9 (-4.7, 20.1) | 2.0 (-5.4, 10.0) |
| M*i*BP | -5.4 (-14.3, 4.3) | -1.1 (-7.3, 5.5) | -4.7 (-11.0, 2.0) | 0.4 (-5.9, 7.1) | -1.0 (-5.0, 3.1) |
| M*n*BP | **-15.6 (-28.2, -0.9)^*^** | **10.2 (-0.9, 22.5)^**^** | -1.1 (-12.9, 12.2) | **10.3 (-0.9, 22.6)^**^** | 0.5 (-6.5, 8.0) |
| MB*z*P | 0.8 (-6.6, 8.7) | 5.3 (0.5, 10.4)^*^ | 1.0 (-4.5, 6.8) | 3.7 (-1.3, 8.9) | 2.2 (-0.9, 5.4) |
| MECPP | -2.1 (-6.9, 2.8) | -0.7 (-3.8, 2.5)^**^ | **-4.4 (-7.4, -1.3)^*^** | 0.5 (-2.8, 3.8) | -1.0 (-3.1,1.0) |
| MEHHP | -5.4 (-11.3, 0.9) | 0.9 (-3.4, 5.4) | -3.1 (-7.5, 1.6) | 1.0 (-3.3, 5.7) | 0.5 (-2.3, 3.4) |
| MEOHP | -5.4 (-11.1, 0.7)^**^ | 0.4 (-4.0, 5.0) | -3.3 (-7.9, 1.4) | 1.7 (-2.8, 6.4) | 0.4 (-2.5, 3.4) |
| MEHP | -1.8 (-10.3, 7.6) | 3.0 (-2.8, 9.2) | **-6.5 (-12.1, -0.5)^*^** | 3.4 (-2.4, 9.4) | -1.7 (-5.4, 2.1) |
| ∑_4_DEHP | -2.8 (-8.1, 2.9) | -0.4 (-4.0, 3.5) | **-4.7 (-8.2, -1.0)^*^** | 1.0 (-2.8, 4.8) | -1.0 (-3.3, 1.4) |
| ∑_3_DEHP | -2.9 (-8.0, 2.4) | -0.6 (-4.0, 3.0) | **-4.2 (-7.6, -0.8)^*^** | 0.8 (-2.8, 4.4) | -0.8 (-3.0, 1.5) |
| ∑DBP | **-12.2 (-23.4, 0.7)^**^** | 4.6 (-4.6, 14.7) | **-8.5 (-17.3, 1.1)^**^** | 6.5 (-2.8, 16.8) | -2.0 (-7.6, 4.1) |

Blount, B.C., Milgram, K.E., Silva, M.J., Malek, N.A., Reidy, J.A., Needham, L.L., Brock, J.W., 2000b. Quantitative detection of eight phthalate metabolites in human urine using HPLC-APCI-MS/MS. Anal Chem 72, 4127-4134.

Carrillo, J.D., Martínez, M.P., Tena, M.T., 2008. Determination of phthalates in wine by headspace solid-phase microextraction followed by gas chromatography-mass spectrometry. Use of deuterated phthalates as internal standards. Journal of chromatography. A 1181, 125-130.

Hays, S.M., Aylward, L.L., Blount, B.C., 2015. Variation in urinary flow rates according to demographic characteristics and body mass index in NHANES: potential confounding of associations between health outcomes and urinary biomarker concentrations. Environ Health Perspect 123, 293-300.

Langlois, E., Leblanc, A., Simard, Y., Thellen, C., 2012. Accuracy investigation of phthalate metabolite standards. J Anal Toxicol 36, 270-279.

Liu, W., 2008. Determination of sub-ppb level of phthalates in water by auto-SPME and GC-MS. Agilent Technologies.

Servaes, K., Voorspoels, S., Lievens, J., Noten, B., Allaerts, K., Van De Weghe, H., Vanermen, G., 2013. Direct analysis of phthalate ester biomarkers in urine without preconcentration: method validation and monitoring. J Chromatogr A 1294, 25-32.

Silva, M.J., Malek, N.A., Hodge, C.C., Reidy, J.A., Kato, K., Barr, D.B., Needham, L.L., Brock, J.W., 2003. Improved quantitative detection of 11 urinary phthalate metabolites in humans using liquid chromatography-atmospheric pressure chemical ionization tandem mass spectrometry. J Chromatogr B Analyt Technol Biomed Life Sci 789, 393-404.

Silva, M.J., Samandar, E., Preau, J.L., Jr., Reidy, J.A., Needham, L.L., Calafat, A.M., 2007. Quantification of 22 phthalate metabolites in human urine. J Chromatogr B Analyt Technol Biomed Life Sci 860, 106-112.

US EPA, United States Environmental Protection Agency, 2012. Federal Register, Definition and procedure for determination of the method detection limit.

Wang, H.X., Wang, B., Zhou, Y., Jiang, Q.W., 2013. Rapid and sensitive analysis of phthalate metabolites, bisphenol A, and endogenous steroid hormones in human urine by mixed-mode solid-phase extraction, dansylation, and ultra-performance liquid chromatography coupled with triple quadrupole mass spectrometry. Anal Bioanal Chem 405, 4313-4319.
